# A chlorinated diketopiperazine antibiotic targets *Mycobacterium tuberculosis*

**DOI:** 10.1128/aac.00369-25

**Published:** 2025-07-31

**Authors:** Libang Liang, Jeffrey Quigley, Monique Theriault, Akira Iinishi, Rachel Bargabos, Madeleine Morrissette, Megan Ghiglieri, Tom Curtis, Rachel Corsetti, Sangkeun Son, Bishwarup Sarkar, Kim Lewis

**Affiliations:** 1 Department of Biology, Antimicrobial Discovery Center, Northeastern University 1848, Boston, Massachusetts, USA; Bill & Melinda Gates Medical Research Institute, Cambridge, Massachusetts, USA

**Keywords:** tuberculosis, antimicrobial agents, DNA gyrase, drug discovery

## Abstract

We describe a novel macrocyclic peptide, speirobactin, produced by *Photorhabdus temperata* that selectively kills *Mycobacterium tuberculosis*. A nonribosomal peptide synthase containing two linear modules codes for the synthesis of speirobactin. The biosynthetic operon contains a pentapeptide-repeat protein as a resistance gene. Genomic analysis of speirobactin-resistant mutants of *M. tuberculosis* led to the identification of DNA gyrase as the molecular target. The mutations were recreated and show that DNA gyrase is the only target. Transcriptome analysis of *M. tuberculosis* treated with antibiotics shows that speirobactin clusters close to fluoroquinolones, supporting its action against the DNA gyrase.

## INTRODUCTION

*Mycobacterium tuberculosis* is the leading cause of death from a single infectious agent according to the World Health Organization (https://www.who.int/news-room/fact-sheets/detail/tuberculosis). The standard of care for treating drug-susceptible *M. tuberculosis* requires an intensive treatment with four antibiotics—rifampin, isoniazid, pyrazinamide, and ethambutol for 8 weeks, followed by 18 weeks of extended treatment using isoniazid and rifampin. In 2021, results of a phase III clinical trial supported a 4-month treatment using rifapentine, and moxifloxacin was noninferior to the standard 6-month treatment (1). Following this study, the Centers for Disease Control and Prevention issued an interim guidance to recommend the 4-month rifapentine-moxifloxacin regimen for treating patients aged over 12 with drug-susceptible pulmonary tuberculosis. The prolonged treatment and patient noncompliance have given rise to drug-resistant *M. tuberculosis* strains (2, 3). New antibiotics are needed to combat drug-resistant *M. tuberculosis*.

Streptomycin was the first antibiotic able to treat tuberculosis. Another natural product compound, rifampicin (or rifabutin), is still an important component of the treatment regimen. Yet, resistance to the first-line antibiotics, such as rifampicin, is a major concern for tuberculosis. In search of additional compounds to treat tuberculosis, we introduced differential screening and focused on untapped sources of secondary metabolites. A very large background of toxic and known compounds is a formidable bottleneck for antibiotic discovery. Screening, in parallel, against *M. tuberculosis* and a different species such as *Staphylococcus aureus* resolves this bottleneck. Only compounds acting selectively against *M. tuberculosis* are considered, and with selective activities, these compounds cannot be generally toxic. Selectivity against a particular species of bacteria also suggests lack of action against eukaryotic cells (4). This approach, coupled with a screen of uncultured bacteria, has led to the discovery of lassomycin, an inhibitor of the mycobacterial ClpP1P2C1 protease from *Lentzea kentuckyensis* sp., and amycobactin, an inhibitor of mycobacterial SecY protein exporter produced by *Amycolatopsis* sp. (5, 6). Searching for untapped sources of novel compounds, we have been screening *Photorhabdus*, symbionts of nematode gut microbiome. The entomopathogenic nematodes infect insect larvae and release *Photorhabdus* that make antimicrobial compounds to fend off competitors. These compounds should be nontoxic to the nematode. This approach led to the discovery of darobactins and dynobactins, inhibitors of the BamA outer membrane chaperone of gram-negative bacteria (7, 8); evybactin, a DNA gyrase inhibitor produced by *Photorhabdus* sp. smuggled into *M. tuberculosis* cells by the transporter BacA (9) as well as 3′-amino-3′-deoxyguansine, a prodrug interfering transcription in *E. coli* (10). In this study, a differential screen of *Photorhabdus* resulted in the discovery of speirobactin, an inhibitor of DNA gyrase that acts selectively against *M. tuberculosis*.

## RESULTS AND DISCUSSION

### Bioassay-guided isolation and characterization of speirobactin

A small set of 17 *Photorhabdus* isolates (Table S1) was fermented in various liquid media to access natural products. The fermentations were extracted to test against *Mycobacterium tuberculosis* in a fluorescence assay using microtiter plates and counter-screen against *Staphylococcus aureus* in an agar diffusion assay. The extracts produced 26 hits against *M. tuberculosis* (Table S2), and after eliminating highly active hits against *S. aureus*, only two extracts from *P. temperata* He86 and *P. temperata* K122 were followed up. The two extracts had over 90% inhibition in the *M. tuberculosis* fluorescence assay, while their activities against *S. aureus* were relatively weak. One fermentation medium was chosen for each of the two producers for further scale-up (Table S3). *M. tuberculosis* assay-guided fractionation was conducted on the scale-up extracts using C18 column chromatography and subsequently high-performance liquid chromatography (HPLC) with an UV detector. The *M. tuberculosis* inhibiting compounds were isolated by HPLC-UV.

The purified compounds were analyzed by liquid chromatography-high-resolution mass spectrometry (LC-HRMS). Compounds from both *P. temperata* He86 and *P. temperata* K122 shared the same HPLC retention time and the same exact mass with an *m/z* [M + H]^+^ of 376.0801, suggesting the same active compound was produced by both strains. There was a characteristic isotopic ion with an *m/z* of 378.0772 at about a third of the peak intensity, suggesting the molecule contained one chlorine. With one chlorine in the molecule, there was only one molecular formula C_16_H_14_N_5_O_4_Cl under 5 ppm mass deviation. Therefore, the molecular formula C_16_H_14_N_5_O_4_Cl was assigned for the neutral molecule, which we named speirobactin. Another 10 L scale-up using the producer *P. temperata* He86 followed by extraction and purification afforded 3 mg of the purified speirobactin for structure elucidation.

**Fig 1:**
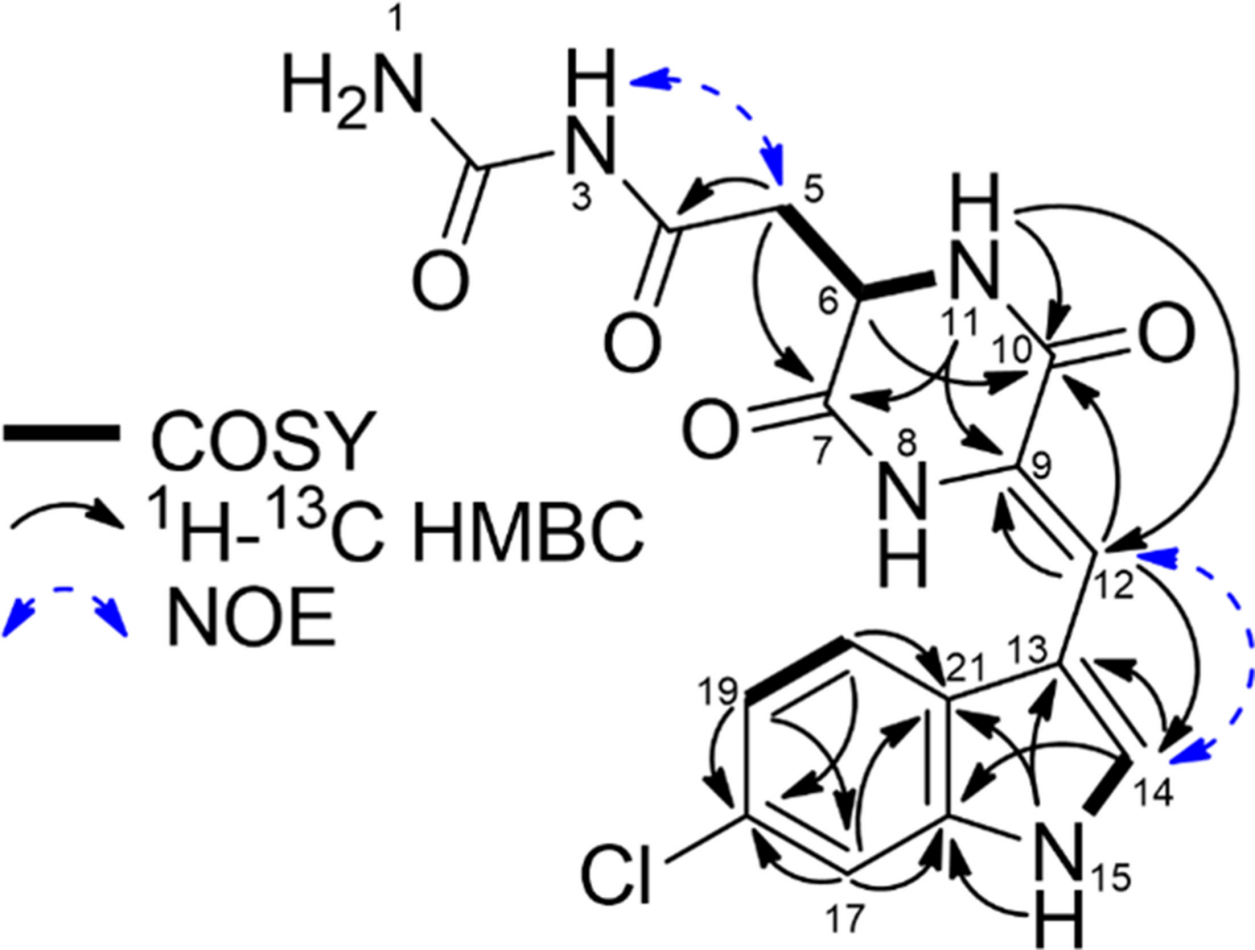
Key 2D NMR correlation of speirobactin in DMSO-*d_6_*.

A combination of 1D and 2D nuclear magnetic resonance (NMR) analysis (Fig. 1), including ^1^H, ^13^C, COSY (correlation spectroscopy), ^1^H-^13^C HSQC (heteronuclear single quantum coherence), ^1^H-^13^C HMBC (heteronuclear multiple bond correlation), TOCSY (total correlation spectroscopy), and NOESY (nuclear overhauser effect spectroscopy), established the diketopiperazine backbone structure of speirobactin consisting of a tryptophan moiety and an asparagine moiety (Fig. S1 to S7; Table S4). The amine proton H-15 has HMBC correlations to C-16, C-13, and C-21, and the aromatic proton H-14 has HMBC correlations to C-21, C-13, and C-16, establishing the five-member ring in the tryptophan moiety. Aromatic protons H-17 and H-20 showed HMBC correlations to C-21, and H-17 showed the HMBC correlation to C-16, suggesting the three aromatic protons H-17, H-19, and H-20 were in an aromatic system connected to the five-member ring. A COSY correlation between H-19 and H-20 suggested C-19 was connected to C-20. H-17, H-19, and H-20 showed HMBC correlations to C-18, the last carbon in the six-member ring of tryptophan. Although H-19 showed an HMBC correlation to C-17, there was a lack of TOCSY correlations between H-17 and H-19, which indicated C-18 was attached to a heteroatom. The carbon chemical shift of C-18 could not support an oxygen or nitrogen substitution; based on the molecular formula, a chlorine was assigned at the C-18 position. H-12 showed an HMBC correlation to C-14, and H-12 and H-14 had a nuclear overhauser effect (NOE) correlation, establishing the H-12 as the β-proton in the tryptophan. The presence of the strong NOE correlation between H-12 and H-14 and the absence of the NOE correlation between H-12 and NH-8 established the *Z*-configuration of the double bond. Additionally, the *Z*-configuration was deduced by the downfield proton chemical of H-12 (*δ*_H_ 6.92) shielded by the carbonyl at C-10, which had been reported in multiple structurally related diketopiperazines (11, 12). H-12 also showed HMBC correlations to C-9 and the carbonyl carbon C-10, suggesting C-9 was the α-carbon of the tryptophan. A double bond was assigned between C-9 and C-12 based on their chemical shifts, and only one proton was attached to C-12.

Another amide proton H-11 showed HMBC correlations to C-10, C-9, and the asparagine carbonyl C-7. COSY correlations between H-11 and H-6 as well as between H-5 and H-6 suggested H-5, H-6, and H-11 were in the same spin system. H-5 as the β-proton showed an HMBC correlation to the carbonyl C-7 and another carbonyl C-4. An NOE correlation between H-5 and the amide proton H-3 established the assignment of the asparagine moiety. A carbon signal at 154.1 ppm was observed in the ^13^C spectrum, which was the last unassigned carbon from the molecular formula. Together with the last unassigned nitrogen, a carbamide moiety was proposed to attach to the side chain of the asparagine. MS/MS analysis of the speirobactin [M + H]^+^ ion 376.08 resulted in ions with m/z of 359.04, 333.15, and 316.06, which corresponded to loss of NH_2_, CONH_2_, and NH_2_CONH_2_ from the asparagine side chain (Fig. S8), confirming the carbamide assignment. Finally, the connection between the carbonyl C-7 and the tryptophan N-terminal was assigned to complete the degree of unsaturation, forming a diketopiperazine core structure.

We noticed the structure of speirobactin contained a chiral center at the asparagine moiety. The stereochemistry was analyzed with a modified Marfey’s method. The analysis showed the hydrolyzed speirobactin contained both D- and L-aspartic acid (Fig. S10). Furthermore, the racemic mixture of speirobactin was separated using chiral HPLC (Fig. S9), and the MIC for (6R)-speirobactin was 0.25 µg/mL while (6S)-speirobactin was 4 µg/mL. We also acknowledge that the activity from (6S)-speirobactin could come from the contamination of (6R)-speirobactin.

The speirobactin MIC against *M. tuberculosis* was 0.25 µg/mL and 16 µg/mL against the counter-screen organism *S. aureus*. This shows that speirobactin is a highly potent and selective antibiotic acting against *M. tuberculosis*. Speirobactin had low or moderate activity against human pathogens and gut commensals, confirming its selectivity against *M. tuberculosis* (Table 1). Notably, while speirobactin was inactive against wild-type *E. coli*, it was highly potent against *E. coli* WO153, a strain with a defective permeability barrier, deficient in the production of lipopolysaccharide and lacking TolC, a porin for docking Multi-Drug Resistance (MDR) pumps (13). Speirobactin was not active against the tested mammalian cells (Table 1). We acknowledge that the lack of cytotoxicity could also come from the molecule binding to serum.

**TABLE 1:** Activity of speirobactin against pathogens, commensals, and human cells^*c*^

Organism and strain	Speirobactin concentration (µg/mL)
Pathogenic bacteria (MIC)	
*Mycobacterium tuberculosis* H37Rv	0.25
*Mycobacterium smegmatis* mc^2^155	16
*Borrelia burgdorferi* BbP1286	1.25
*Staphylococcus aureus* HG003	16
*Pseudomonas aeruginosa* PAO1	16
*Klebsiella pneumoniae* ATCC 700603	>128
*Escherichia coli* MG1655	>128
*Escherichia coli* WO153	0.25
*Acinetobacter baumannii* ATCC17978	>128
Human anaerobic commensals (MIC)	
*Bacteroides fragilis* KLE 2244^*a*^^,*b*^	8
*Bacteroides stercoris* KLE 2537^*a*^^,*b*^	16
*Veillonella ratti* KLE 2365^*a*^^,*b*^	32
*Lactobacillus reuteri* LTH5448^*a*^	>128
*Clostridium perfringens* KLE 2523^*a*^^,*b*^	32
*Clostridium tertium* KLE 2303^*a*^^,*b*^	8
*Enterococcus faecalis* KLE 2341^*a*^^,*b*^	4
Cell lines (IC_50_)	
Hek293	>128
Fadu	>128
HepG2	>128

^*a*^ Cultured under anaerobic conditions.

^*b*^ Human stool isolates; KLE, Kim Lewis laboratory collection.

^*c*^ MIC determination was performed in triplicate. Rifampicin was tested as a control for *M. tuberculosis*, with an MIC of 0.125 µg/mL.

### Biosynthetic gene cluster of speirobactin

**Fig 2:**
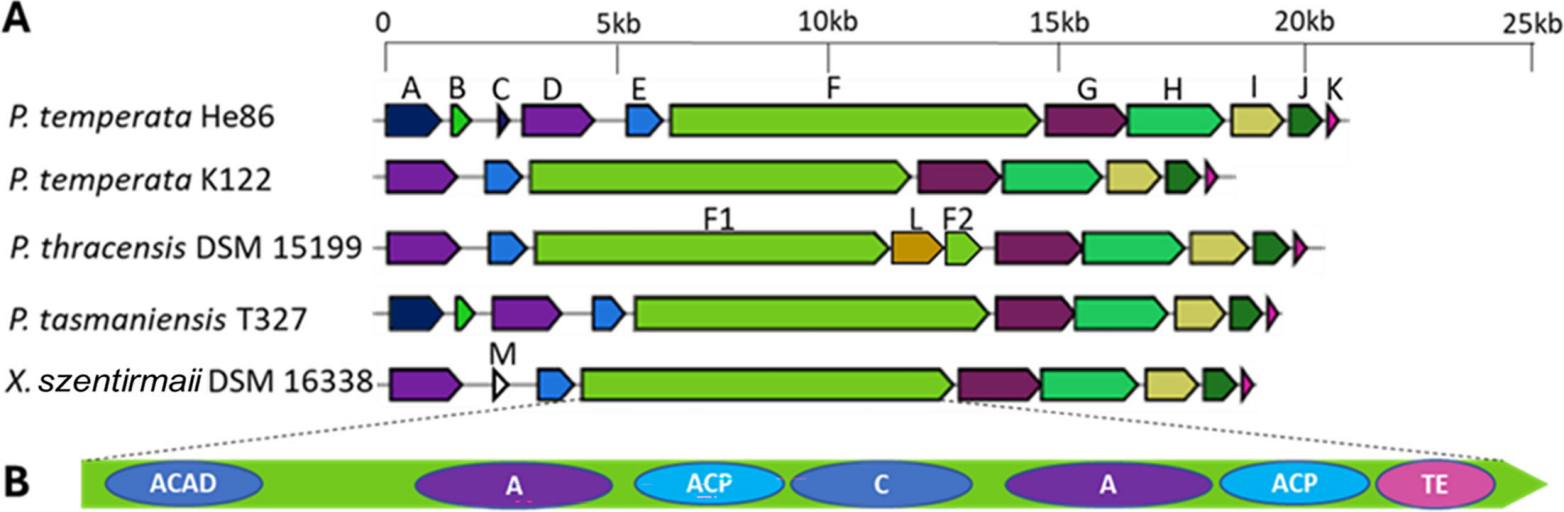
Biosynthetic gene cluster of speirobactin: (**A**) alignment of biosynthetic genes across *Photorhabdus* and *Xenorhabdus* strains, and (**B**) domains identified in the NRPS gene in the BGC. E, pentapeptide-repeat protein.

Genome sequencing and assembly of the producers *P. temperata* He86 and *P. temperata* K122 resulted in two genomes both at 5.6 M bp. AntiSMASH 7.0 (14) was used to profile biosynthetic gene clusters (BGCs) in the two genomes, and 26 and 22 BGCs were identified for *P. temperata* He86 and *P. temperata* K122, respectively. Based on the elucidated structure of speirobactin, its chemical backbone contains tryptophan and asparagine. We started to focus on nonribosomal peptide synthetases (NRPSs) and narrowed down to specific pathways that incorporate two amino acids. In this way, the expected NRPS should contain two linear modules. All BGCs containing NRPSs from the AntiSMASH output were carefully examined. An operon spanning 21 kb was identified from *P. temperata* He86 containing an NRPS with 2 linear modules and 11 open reading frames. Although AntiSMASH did not pick up the loading domain in the first module of the NRPS, the NCBI Conserved Domain tool (15) was able to locate an acyl-CoA dehydrogenase (ACAD) in the upstream region of the first module. Therefore, the NRPS gene (gene F) in the operon contained seven identified linear modules: acyl-CoA dehydrogenase, adenylation (A), acyl carrier protein (ACP), condensation (C), adenylation, acyl carrier protein, and thioesterase (TE) (Fig. 2 and Table 2). The ACP module is commonly involved in loading the initial amino acid, while the TE domain is commonly involved in the final step of NRPS biosynthesis (16–18). In between the ACAD and TE, there were two linear NRPS modules. This gene matched our initial search criteria for an NRPS incorporating two amino acids. The ACAD was proposed to load the initial amino acid, which was recognized by the first adenylation domain and transferred by the first ACP to the condensation domain in the second module. In the second module, the adenylation domain specifically recognized and bonded to a second amino acid, which underwent condensation with the first amino acid to form a dipeptide. The dipeptide was eventually carried by the second ACP to the TE domain, where the molecule got hydrolyzed and cleaved off from the NRPS protein (Fig. 2 and Table 2). A homologous BGC was also found in *P. temperata* K122, without the A, B, and C genes, suggesting the A, B, and C genes were not essential in the biosynthesis of speirobactin. Further analysis with NRPSPredictor2 (19) showed weak substrate specificity prediction for both adenylation domains; the Stachelhaus sequences (20) for the two adenylation domains were identified as DYWQIGFIDK and DIWYVGACSK, both giving low matching scores for substrate specificity for tryptophan and asparagine. A tryptophan halogenase (gene D) was identified upstream to the NRPS; a carbamoyl transferase (gene G) and an aspartate racemase (gene J) were identified downstream. The isolated speirobactin contains both (6S)- and (6R)-stereoisomers, suggesting the corresponding adenylation domain incorporating both L- and D-aspartate generated by the aspartate racemase (gene J). (6R)-speirobactin utilizing D-aspartate was the more active stereoisomer, suggesting the racemase was part of the biosynthetic machinery to produce D-aspartate. Therefore, we hypothesize that the 21-kb NRPS BGC is responsible for the production of speirobactin.

**TABLE 2:** Biosynthetic genes identified from the *Photorhabdus* and *Xenorhabdus* genomes^*a*^

Biosynthetic enzyme	Nucleotide location	GC content	Number of amino acids	Proposed function in speirobactin biosynthesis^*b*^	Homologous protein from NCBI, organism, identity%/coverage%, accession no.
A	289,514–290,719	39%	401	Transporter	MFS transporter, *Photorhabdus tasmaniensis*, 99/100, WP_133816297.1
B	290,986–291,420	33%	144		Hypothetical protein, *Photorhabdus tasmaniensis*, 100/100, WP_133816296.1
C	292,038–292,235	37%	65		No significant similarity found
D	292,556–294,112	36%	518	Tryptophan halogenase	Tryptophan halogenase, *Photorhabdus thracensis*, 96/96, AKH65769.1
E	294,859–295,614	31%	251	Self-resistance	Pentapeptide repeat-containing protein, *Photorhabdus kayaii*, 87/100, NDL14150.1
F	295,800–303,953	37%	2,717	Nonribosomal peptide synthetase	Amino acid adenylation domain-containing protein, *Xenorhabdus* sp. 42, 76/100, MBD2820043.1
G	304,120–305,892	40%	590	Carbamoyl transferase	Carbamoyl transferase, *Photorhabdus heterorhabditis*, 91/100, KOY60149.1
H	305,921–308,053	39%	710		SGNH/GDSL hydrolase family protein, *Photorhabdus cinerea*, 84/100, WP_166305217.1
I	308,184–309,368	37%	394	Transporter	MFS transporter, *Xenorhabdus* sp. M, 82/99, MBD2801864.1
J	309,460–310,194	43%	244	Aspartate racemase	Aspartate/glutamate racemase family protein, *Xenorhabdus* sp. CUL, 86/96, MBD2791922.1
K	310,307–310,573	46%	88		Hypothetical protein, *Photorhabdus thracensis*, 97/98, AKH65770.1
F1	1,311,013–1,318,398	36%	2,461	Truncated NRPS (ACAD-A-ACP-C-A-ACP)	Hypothetical protein, *P. laumondii* subsp. *clarkei*, 80/99, RAW88993.1
L	1,318,422–1,319,624	50%	400	Transposase	IS256 family transposase, *P. luminescens*, 99/100, PQQ39455.1
F2	1,319,722–1,320,480	37%	252	Truncated NRPS (TE)	Hypothetical protein, *P. thracensis*, 100/100, AKH62816.1
M	2,189,852–2,190,118	43%	88	Transposase	IS5 family transposase, *P. kayaii*, 80/94, NDL28030.1

^*a*^ Note: Genes A–K were from the *P. temperata* He86 circular genome, genes F1, L, and F2 were from *P. thracensis* DSM 15199, and gene M was from *X. szentirmaii* DSM 16338.

^*b*^ The empty cells in “proposed function in speirobactin biosynthesis” indicate the respective gene functions are unclear.

We also found homologous BGCs in *P. thracensis* DSM 15199, *P. tasmaniensis* T327, and *Xenorhabdus szentirmaii* DSM 16338 based on their genomes in the NCBI database (Table 2). To confirm the link between the identified BGC and speirobactin, we acquired these strains and analyzed their fermentation extracts by UPLC-HRMS/MS. We were able to identify speirobactin from extracts of *P. tasmaniensis* T327 and *X. szentirmaii* DSM 16338 based on exact mass, MS^2^ pattern, and retention time but not from *P. thracensis* DSM 15199. A detailed analysis of the NRPS gene in *P. thracensis* DSM 15199 showed an insertion sequence (IS) between the ACP domain of the second module and the TE domain. The insertion sequence (gene L in Fig. 2) has high homology to IS256 family transposase, an IS widespread in enterococci and staphylococci (21). We reason that the truncation of the NRPS gene in *P. thracensis* DSM 15199 by an IS resulted in the lack of speirobactin production. We identified gene M, an IS5 family transposase in the homologous BGC of *X. szentirmaii* DSM 16338 (Fig. 2). The absence of genes A, B, and C in the speirobactin-producing *X. szentirmaii* DSM 16338 suggests these three genes are not essential for its production. The speirobactin BGC is restricted to *Photorhabdus* and *Xenorhabdus*, based on the search of NCBI databases. However, several genes of this BGC have unusually low GC content, 31%–33%, compared to 40% for the rest of the genome. This operon was probably horizontally acquired (or assembled from horizontally acquired components) from an unknown microorganism.

We noticed that the BGC of speirobactin contained a gene (E) coding for a pentapeptide repeat-containing protein (PRP) (Table S5). The best-studied member of this family is QnrA that confers resistance to fluoroquinolones. QnrA and its homologs are carried on transmissible plasmids and are present in clinical isolates of *Klebsiella pneumoniae, E. coli,* and other Enterobacteriaceae. The mechanism of resistance is highly unusual. QnrA folds into a quadrilateral β-helix structure that mimics DNA, which enables it to bind and sequester DNA gyrase, the target of fluoroquinolone antibiotics. Normally, diminishing the level of a target leads to increased antibiotic susceptibility. However, fluoroquinolone antibiotics act by corrupting their target rather than inhibiting its function. Fluoroquinolones stabilize the transient gyrase/DNA cleavage complex, leading to double-strand break and cell death. Diminishing the level of active gyrase will then result in partial resistance. Notably, a PRP protein, McbG, was identified as an immunity factor in an *E. coli* operon coding for Microcin B17, an antimicrobial peptide targeting the DNA gyrase (22).

A Qnr homolog MfpA (mycobacterial fluoroquinolone resistance protein) was also reported in *M. tuberculosis, M. smegmatis, M. bovis, M. ulcerans,* and *M. avium* (23). The closest homologs of gene E in the speirobactin BGC were restricted to *Photorhabdus* and *Xenorhabdus* in the NCBI database. We reason that the *Photorhabdus* PRP is a self-resistance gene, and the target of speirobactin is the DNA gyrase.

### Molecular target

In order to identify the molecular target of speirobactin, *M. tuberculosis* cells were plated on a medium containing a high concentration of compound, 125 µg/mL. Resistant colonies appeared with a frequency of 1.5 × 10^−8^. Whole-genome sequencing of four stable speirobactin-resistant mutants was performed by Illumina sequencing. All mutants carried mutations in the DNA gyrase; no other mutations were shared among these strains (Table 3; consolidated mutations in Table S6; all mutations in wild-type and speirobactin-resistant strains in Supplemental material B). In order to validate the mutations, GyrA D89G and Q101H were recreated in wild-type *M. tuberculosis* using single-strand recombineering. Both mutations conferred resistance to speirobactin at levels over 128 µg/mL. We were not able to recreate the R128K mutation in the fresh background of the wild type. We hypothesized R128K was essential to the survival of *M. tuberculosis,* and mutation at this site impacted the vitality of the strain. We have listed all mutations identified in the R128K mutant strains in Supplemental material B, and while no clear compensatory mutations were observed, we cannot definitively rule out the possibility that some of the additional mutations may play a compensatory role.

**TABLE 3:** DNA gyrase mutation sites in speirobactin-resistant *M. tuberculosis*

Mutant no.	Position	Mutation	Annotation	Gene	MIC (µg/mL)
1	7567	A→G	D89G(GAC→GGC)	*gyrA*	>128
2	7604	G→C	Q101H(GAC→GGC)	*gyrA*	32^*a*^
3	7567	A→G	D89G(GAC→GGC)	*gyrA*	>128
4	7684	G→A	R128K(AGG→AAG)	*gyrA*	>128

^*a*^ MIC measurement from strain obtained in the mutant selection experiments; when the same mutation was created from wild-type Mtb by single-stranded recombineering, MIC was >128 µg/mL.

According to the crystal structure of *M. tuberculosis* GyrA (*Mt*Gyr59) (24), D89, Q101, and R128 are invariant sites close to the active site of Y129; mutations in these sites likely affect the activity of the enzyme. These results confirm GyrA as the target of speirobactin. Significantly, very high resistance of mutants shows that speirobactin does not have off-target activity.

Fluoroquinolones are the most important gyrase-targeting drugs for clearing serious *M. tuberculosis* infections in the clinic. Most bacteria have two type II topoisomerases: DNA gyrase and topoisomerase IV. Interestingly, *M. tuberculosis* only has a DNA gyrase as the sole type II topoisomerase, and it is commonly assumed to perform the function of both gyrase and topoisomerase IV, i.e., relaxing the supercoiling and unlinking the replicated chromosomes.

**Fig 3:**
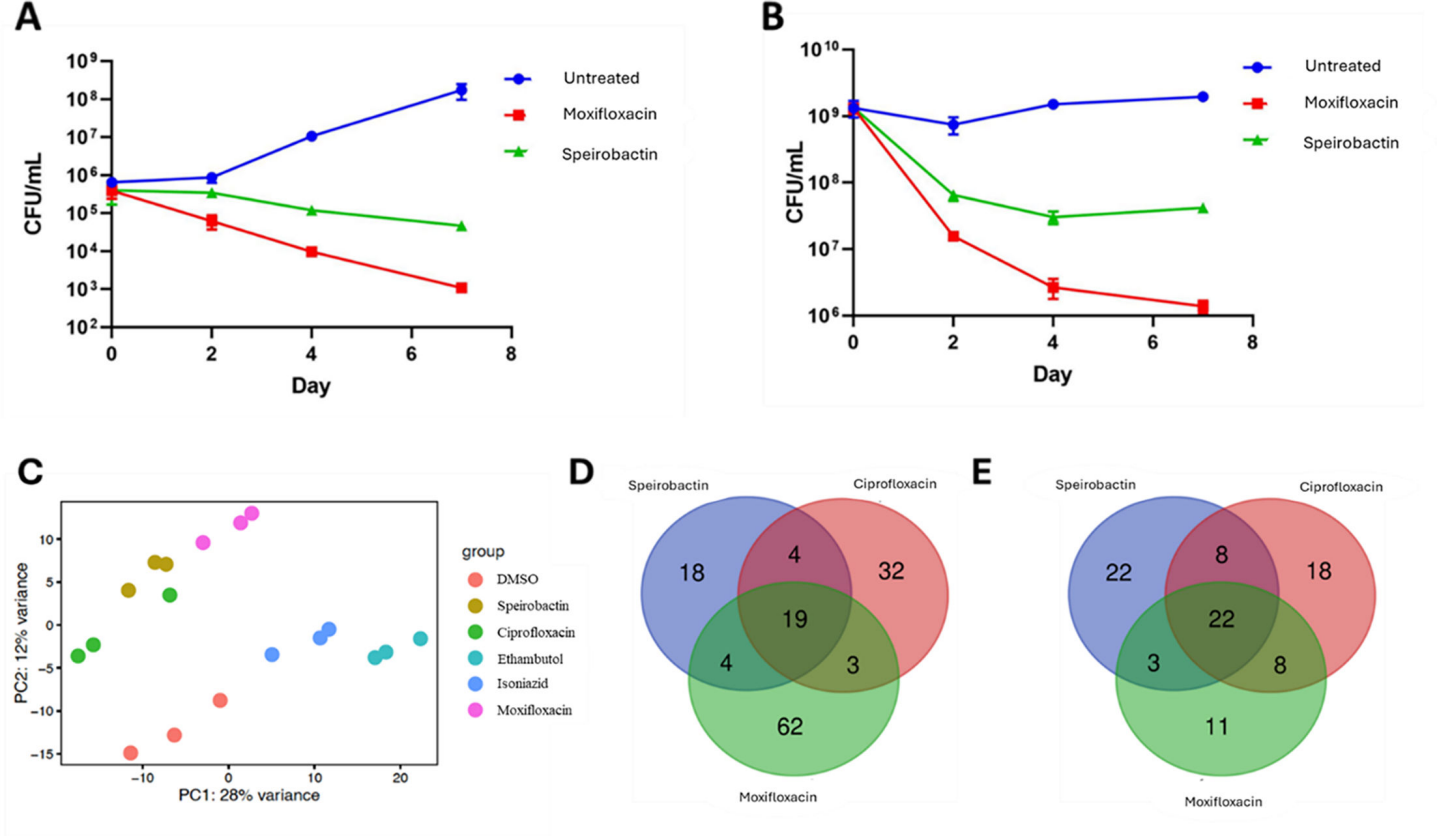
Action of speirobactin against *M. tuberculosis*. (**A**) Killing of *M. tuberculosis* by speirobactin and moxifloxacin in mid-exponential phase (*N* = 3). (**B**) Killing of *M. tuberculosis* by speirobactin and moxifloxacin stationary-phase *M. tuberculosis* (*N* = 3). Cultures were treated with 10× MIC (MIC for both compounds is 0.125 µg/mL). The experiment was performed with biological triplicates and error bars representing standard deviations. (**C**) Principal component analysis of RNA-seq data where *M. tuberculosis* was treated with 2× MIC of speirobactin, moxifloxacin, ciprofloxacin, isoniazid, and ethambutol for 4 hours (detailed transcriptome analysis in Supplemental material C) (*N* = 3). (**D**) Venn diagram for genes upregulated shared by speirobactin, moxifloxacin, and ciprofloxacin (detailed gene descriptions in Supplemental material D). (**E**) Venn diagram for genes downregulated shared by speirobactin, moxifloxacin, and ciprofloxacin (detailed gene descriptions in Supplemental material D).

We tested the bactericidal activity of speirobactin and compared it to moxifloxacin that is used to treat tuberculosis, in exponential and stationary phase cells. Speirobactin was able to kill both exponential- and stationary-phase *M. tuberculosis*, although killing was somewhat weaker than moxifloxacin (Fig. 3A and B). Notably, starting at day 2, killing kinetics were linear for exponential cells with both moxifloxacin and speirobactin. Stationary cells are especially hard to kill, yet speirobactin decreased their burden by over a log; after day 4, killing reaches a plateau, typical biphasic kinetics showing a subpopulation of surviving persisters. Given the slow growth of the pathogen, killing experiments are sometimes extended beyond 7 days, but in this case, the kinetics provide a fairly clear picture by day 4.

To further understand the mode of action of speirobactin, RNA-seq analyses were performed in *M. tuberculosis* comparing speirobactin, moxifloxacin, ciprofloxacin, and two front-line antibiotics isoniazid and ethambutol. While moxifloxacin and ciprofloxacin are known as DNA gyrase targeting antibiotics, isoniazid and ethambutol are cell wall targeting. In order to prevent a complete halt in transcription but still capture compounds’ transcriptional signature, bacteria were treated at 2× MIC for a short period of 4 hours. Speirobactin clustered in between that of moxifloxacin and ciprofloxacin and away from the cell wall inhibitors and untreated samples (Fig. 3C).

Based on resistance mutations, transcriptome analysis, and the location of a PRP within the speirobactin BGC studies, *gyrA* is predicted to be the molecular target of speirobactin. Expression of *gyrA* (*rv0006*) was not impacted by speirobactin treatment, which is consistent with moxifloxacin and ciprofloxacin (detailed transcriptome analysis in Supplemental material C). However, there was a noted upregulation of genes involved in DNA repair that was shared between these three treatment groups (Fig. 3D; Table S7, detailed gene descriptions in Supplemental material D). Downregulated genes shared between speirobactin and the fluoroquinolones were involved with various metabolic processes and did not show an obvious transcriptional signature (Fig. 3E; Table S8, detailed gene descriptions in Supplemental material C).

In addition to the differentially expressed genes that were shared between speirobactin and the fluoroquinolones, there were 22 downregulated genes and 18 upregulated genes that were unique to speirobactin. Analyses via STRING showed no significant enrichment detected in the upregulated genes, and minor signatures related to transport (in particular, the Mce2 operon) detected in the downregulated genes (Fig. S13 and S14).

We further tested the cross-resistance effects between speirobactin and moxifloxacin. Speirobactin-resistant mutants were susceptible to moxifloxacin with their MIC values close to the wild-type MIC (Table 4). However, the behavior of moxifloxacin-resistant mutants was more complex and depended on the site of mutation: the G88C mutant was fully resistant to speirobactin, while D94N was susceptible to it. These results show that although both compounds targeted DNA gyrase, their mode of action is likely to be different.

**TABLE 4:** Cross-resistance analysis between moxifloxacin and speirobactin^*b*^

Strain	Gyrase mutation	MIC (µg/mL) for moxifloxacin	MIC (µg/mL) for speirobactin
Wild type^*c*^		0.125	0.125^*a*^
Moxifloxacin-resistant mutants	G88C (GGC→TGC)	4	>128
	D94N (GAC→AAC)	2	0.25
Speirobactin-resistant mutants	D89G (GAC→GGC)	0.25	>128
	Q101H (GAC→GGC)	0.125	>128

^*a*^ The MIC of speirobactin against wild-type *M. tuberculosis* was 0.125 µg/mL in this experiment using serially diluted speirobactin. It is noted that in the experiment tested against a panel of pathogens in Table 1, the MIC was 0.25 µg/mL. The difference is within the twofold intrinsic variation of MIC determination.

^*b*^ The moxifloxancin-resistant mutatns and the speirobactin-resistant mutants were further tested against speirobactin and moxifloxacin, respectively.

^*c*^ The wild type genomic sequences are used as the control for mutation analysis. Therefore, the “gyrase mutation” cell is left empty.

### Conclusion

Screening of natural products produced by nematode symbiont *Photorhbabdus* spp. using a bioassay-guided fraction approach resulted in the identification of an antitubercular compound speirobactin. Its potential biosynthetic gene cluster was identified computationally. The gene cluster was found in multiple *Photorhabdus* and *Xenorhabdus* spp. Sequencing *M. tuberculosis*-resistant mutants suggests the molecular target is DNA gyrase. Furthermore, a gene encoding a PRP was identified in the biosynthetic gene cluster, which belongs to a protein family conferring resistance to fluoroquinolones. An RNA-seq experiment was conducted to investigate the mode of action of speirobactin compared to standard-of-care *M. tuberculosis* medicines. A principal component analysis of upregulated genes showed speirobactin replicates were clustered close to fluoroquinolones (ciprofloxacin and moxifloxacin), distinctly away from isoniazid and ethambutol and nontreated controls. RNA-seq experiments showed that speirobactin and the two fluoroquinolones shared upregulated genes for DNA repair, supporting the proposed molecular target as DNA gyrase. We also provided cross-resistance analysis of speirobactin and moxifloxacin. Further *in vivo* PK/PD studies will be needed to evaluate the pharmaceutic potential of speirobactin.

## MATERIALS AND METHODS

### Screening conditions

A total of 17 *Photorhabdus* strains (Table S1) were fermented in 10 mL in 50 mL Falcon tubes using 15 nutritionally diverse media (Table S3) at 28°C, and 200 rpm and 1 mL of the fermentation were sampled on day 2, day 5, and day 8. The samples were centrifuged at 8,000 × *g*, and the supernatants were transferred into 96-well deep well plates, which were dried under vacuum and resuspended in dimythyl sulfoxide (DMSO) to give 1× and 15× concentrates. To perform the assay, *M. tuberculosis* (H37Rv mc2 763 6020 [ΔlysA ΔpanCD] expressing mCherry [ΔlysA ΔpanCD, pBEN_mCherry kanr 764]) cultures were diluted into fresh medium to a final OD_600_ of 0.003, and 147 µL of the diluted cultures was transferred to a 96-well black, clear-bottom microtiter plate (Corning catalog # 07-200-567) containing 3 µL of 1× or 15× concentrated *Photorhabdus* fermentation supernatant. The assay plates were incubated for 7 days at 37°C and 100 rpm before OD_600_ measurement and fluorescence emission measurement at 610 nm with excitation at 580 nm in a plate reader. The extract was deemed active if there was over 75% growth inhibition compared to nontreatment controls.

The same extracts were tested against *S. aureus* in a disc diffusion assay on agar. Three microliters of the 1× or 15× concentrated *Photorhabdus* fermentation supernatant was spotted on an agar lawn pre-inoculated with log-phase *S. aureus* cultures. The agar plates were incubated at 37°C overnight and observed for zone of inhibition.

### Identification of speirobactin

*Photorhabdus temperata* He86 and *Photorhabdus temperata* K122 were scaled up in 4 L fermentation medium (Bacto peptone 10 g/L, K_2_HPO_4_ 1 g/L, MgSO_4_· 7H_2_O 1 g/L, (NH_4_)_2_SO_4_ 2 g/L, CaCO_3_ 2 g/L, and NaCl 10 g/L) for 5 days at 28°C and 200 rpm. The fermentations were extracted twice with equal volumes of EtOAc for 2 hours. The EtOAc layers were combined, dried *in vacuo,* and fractionated with column chromatography packed with 60 Å SelectraSorb Bulk Sorbent C18 (catalog # 029070-CG). The column was equilibrated with 10 CVs (column volumes) of 5% aq. ACN containing 0.1% formic acid. The crude extracts were loaded and eluted with 5 CVs of 5%, 25%, 50%, and 75% aq. ACN containing 0.1% formic acid, before washing with 10 CVs of 100% ACN containing 0.1% formic acid. Eluants were collected manually in 15 mL fractions and dried *in vacuo*. Each fraction was resuspended in 30 µL 25% DMSO in water, and 3 µL of the suspension was transferred to a 96-well black, clear-bottom microtiter plate for *M. tuberculosis* assay as described above under “Screening conditions”.

The active fractions in the assay were combined and fractionated using an Agilent 1260 Affinity II HPLC system with a Restek Ultra C18 5 µm 250 × 10 mm 100 Å column (catalog # 9174577). The mobile phase consisted of solvent A (H_2_O with 0.1% formic acid) and solvent B (ACN with 0.1% formic acid). The first 2 min were held at 2% solvent B, followed by a linear gradient over 20 min to 100% solvent B, and held at 100% for an additional 10 min. The flow rate was 5 mL/min. A UV detector monitors wavelengths at 220, 280, 310, and 340 nm. A fraction collector system operated in a time-based mode collecting a fraction every 0.3 min. Each fraction was dried *in vacuo* and resuspended in 30 µL 25% DMSO in water, and 3 µL of the suspension was transferred to a 96-well black, clear-bottom microtiter plate for *M. tuberculosis* assay as described above under “Screening conditions”. Pure speirobactin in a fraction was identified as the active compound in the bioassay.

### Scale-up fermentation and preparative HPLC conditions

*Photorhabdus temperata* He86 was scaled up in 20 L using the aforementioned fermentation medium for 5 days at 28°C and 200 rpm. The fermentations were extracted with equal volumes of EtOAc. The EtOAc layers were combined and dried *in vacuo*. The EtOAc extract suspended in DMSO was further loaded onto an open column packed with SelectraSorb Bulk Sorbent C18 (log #029070-CG) pre-conditioned with 2% aqueous ACN. Five column volumes of 2% aqueous ACN were used to remove salts and polar organic compounds. Ten column volumes of ACN were then used to elute the rest of the extract. The ACN eluent was dried *in vacuo*, resuspended in DMSO, then passed through a 0.2 µm filter for HPLC injections. An aliquot of 4.5 mL was injected in an Agilent 1200 series preparative HPLC system with a Primesphere C18-HC 250 × 50 mm, 10 µm particle size column. An isocratic condition with 25% aq. ACN with 0.1% formic acid was used at 75 mL/min. A UV detector monitored wavelengths at 220, 280, and 340 nm. Speirobactin eluted at 23.5 min.

### Structure elucidation

Structure elucidation was conducted by 1D and 2D NMR experiments (^1^H, ^13^C, COSY, NOESY, ^1^H-^13^C HSQC, and ^1^ H-^13^C HMBC) using a Bruker 900 MHz NMR, a Bruker 400 MHz NMR with a cryoprobe, and a Varian 500 MHz NMR. Speirobactin (2 mg) was dissolved in DMSO-*d_6_* in a 5 mm NMR tube, and chemical shifts were referenced to the residue solvent signal (δ_H_ 2.50, δ_C_ 39.52). LC-HRMS/MS analysis was conducted with a LTQ Orbitrap XL Hybrid Ion Trap-Orbitrap Mass Spectrometer (Thermo Scientific) in electrospray ionization positive ion mode coupled with an UltiMate 3000 RSLCnano System chromatography (Dionex). Speirobactin was analyzed with a capillary column (150 mm by ID 75 µm) packed with C18 2.5 mm resin (XSelect CSH C18) at a flow rate of 0.2 µL/min. The mobile phase consisted of solvent A (H_2_O with 0.1% formic acid) and solvent B (ACN with 0.1% formic acid). The first 2 min were held at 5% solvent B and then with a linear gradient increasing solvent B to 50% over 20 min.

**Speirobactin**: pale yellow amorphous solid; UV λmax 234, 290, 342; NMR (700 MHz, DMSO-*d_6_*) see Table S4; HRESIMS [M + H]^+^ *m/z* 376.0801 (calcd for C_16_H_15_N_5_O_4_Cl^+^, 376.0807, Δ = 1.60 ppm).

### Stereochemistry analysis

The stereochemistry at the aspartate moiety of speirobactin was analyzed with a modified Marfey’s method (25). Approximately 150 µg of speirobactin was hydrolyzed with 6 N HCl overnight at 100°C. The hydrolysates of the two compounds were separated into two vials for analysis with both L-FDLA [Nα-(5-fluoro-2,4-dinitrophenyl)-L-leucinamide] and D-FDLA [Nα-(5-fluoro-2,4-dinitrophenyl)-D-leucinamide]. The hydrolysates were dried *in vacuo* and then added with 1 M NaHCO_3_ (20 µL) and the choice of L-FDLA (1% solution in acetone 40 µL) or D-FDLA equivalents. Controls were prepared using L-aspartic acid reacting with both L-FDLA and D-FDLA. The reactions were maintained at 40°C for 1 hour before quenching with 1 M HCl (20 µL). The samples were diluted with another 100 µL of ACN, filtered (0.2 µm), and analyzed by UPLC-HRMS/MS. An aliquot of 2 µL of each sample was injected into the Agilent Poroshell 120 EC-C18 column (3.0 × 150 mm, 2.7 µm, PN: 693975-302) with an Agilent 1260 Affinity HPLC system connected with a PDA detector and an Agilent 6530 QTOF mass spectrometer. The first 2 min were held at 10% solvent B (ACN with 0.1% formic acid) and 90% solvent A (H_2_O with 0.1% formic acid), then followed by a linear gradient at 65% solvent B at 27 min, followed by a linear gradient to 95% B at 27.5 min and held at 95% B until 32 min. The flow rate was 0.3 mL/min. Extracted ion chromatogram was used to monitor the reaction products.

### Chiral separation of speirobactin enantiomers

A sample in DMSO consisting of both enantiomers of speirobactin was injected onto a COSMOSIL CHiRAL Series 5C HPLC column (4.6 × 250 mm, log # 15790-71) using an Agilent 1260 Infinity system monitoring UV absorbance at 342 nm. An isocratic mobile phase at 20% ACN (0.1% formic acid) and 80% H_2_O (0.1% formic acid) was used at 1 mL/min. (6R)-speirobactin eluted at 15.7 min, and (6S)-speirobactin eluted at 18.9 min. The purified enantiomer was subjected to the modified Marfey’s analysis described above in the “Stereochemistry analysis” section.

### Identification of the BGC

An overnight culture of *P. temperata* He86 and *P. temperata* K122 was used to prepare genomic DNA sample using a QIAGEN DNeasy Blood and Tissue Kit following the manufacturer’s instructions. The genomic DNA samples were for Illumina sequencing by Microbial Genome Sequencing Center (MiGS; Pittsburg, PA). Nanopore sequencing was further conducted in-house with a Flongle device (Oxford Nanopore Technologies) following the manufacturer’s instructions. Hybrid genome assembly using the Illumina and Nanopore sequencing data was conducted by Unicycler (26) using default settings. AntiSMASH (14) was used to annotate the two genomes. The homologous genes in the BGC were searched using the NCBI BlastP (15).

### Mtb growth conditions

*M. tuberculosis* MC^2^6020 (*ΔlysA ΔpanCD*) (27) was grown in Difco 7H9 liquid media supplemented with 10% oleic albumin dextrose catalase (OADC), 0.5% glycerol, 0.05% tyloxapol, lysine (80 µg/mL), and pantothenate (24 µg/ml) or 7H10 solid media supplemented with 10% OADC, 0.5% glycerol, lysine (80 µg/mL), and pantothenate (24 µg/mL). For screening, *M. tuberculosis* expressing a plasmid encoding the fluorescent protein mCherry was cultured in 7H9 complete media containing kanamycin and incubated at 37°C and 100 rpm. The mCherry plasmid was constructed by cloning mCherry derived from pCHERRY3 (Addgene plasmid #24659) (28) into pMV261 empty vector. The culture was diluted into fresh medium to a final OD_600_ = 0.003 and aliquoted in a black clear-bottom 96-well plate with bacterial extract. Final dilution of extract was 1:100. The plate was incubated for 7 days at 37°C and 100 rpm, at which point the fluorescence with excitation at 580 nm and emission at 610 nm was measured on a plate reader. The extract was deemed to have activity against *M. tuberculosis* if it had ≥75% growth inhibition when compared to the growth control. The assay was repeated for confirmation of activity.

### Minimum inhibitory concentration

For *M. tuberculosis* assays, a final OD_600_ of 0.003 was obtained by diluting an exponentially growing culture into supplemented 7H9 medium (10% Middlebrook OADC growth supplement [Millipore Sigma]), 5% glycerol, 1% casamino acids, 0.05% tyloxapol, 80 µg mL^−1^ lysine, and 24 µg mL^−1^ pantothenate. Furthermore, 20 µM rifampicin was used as a positive control, and 1% DMSO was used as a negative control for each MIC assay which was conducted. The plates were incubated at 37°C and 100 rpm for 7 days. All batches of purified speirobactin were tested in triplicate for activity against M. *tuberculosis*. For all other microbes, a final OD_600_ of 0.001 was obtained by diluting an exponentially growing culture into BBL Mueller-Hinton II (cation adjusted). The MIC was defined as the lowest concentration of antibiotics with no visible growth.

### Mammalian cytotoxicity

Exponentially growing red fluorescent protein (RFP)-tagged human embryonic kidney HEK239-RFP (GenTarget SC007), FaDu pharynx squamous cell carcinoma (ATCC HTB-43), and HepG2 cells (ATCC HB-8065, in Dulbecco’s Modified Eagle’s medium supplemented with 10% fetal calf serum) were seeded into a 96-well flat bottom plate. After 24 hours of incubation at 37°C, the medium was replaced with fresh medium containing twofold serial dilution of test compounds. After 72 hours of incubation at 37°C, viability was determined by adding Alamar Blue (ThermoFisher) indicator at a final concentration of 10 µg/mL, and IC_50_ was called after 3 hours.

### Mtb time-dependent killing

Exponential culture was prepared by growing *M. tuberculosis* MC^2^6020 (ΔlysA ΔpanCD) to mid-exponential (OD_600_ ~1–1.5), then back diluting to OD_600_ = 0.003. For stationary-phase culture, *M. tuberculosis* grew for 2 weeks to an OD_600_ ~2. Cultures were challenged with 10× MIC of compound at 37°C. At intervals, aliquots were removed, washed once in PBS, and serially diluted and plated on 7H10 media to determine c.f.u. per milliliter.

### Mtb mutant generation

Mutants to speirobactin were generated by plating *M. tuberculosis* on 7H10 medium containing 10×, and 100× MIC of speirobactin. The plates were incubated at 37°C for 3 weeks. Four 10× mutants and four 100× mutants were picked and inoculated into 10 mL 7H9 medium and grown for 2 weeks without selection. Genome sequencing and variant calling were conducted by MiGS (Pittsburg, PA). Mutations in *gyrA* conferring resistance to 100× MIC speirobactin were recreated via single-stranded recombineering as previously described (29) (Table 3). Sequences of oligonucleotides used to make targeted mutations were listed in Fig. S12. Targeted mutations were confirmed via PCR and Illumina sequencing.

### Treatment of Mtb for RNA-seq

*M. tuberculosis* MC^2^6020 (ΔlysA ΔpanCD) was grown in Difco 7H9 liquid media supplemented with 10% OADC, 0.5% glycerol, 0.05% tyloxapol, lysine (80 µg/mL), and pantothenate (24 µg/mL) for 7 days under shaking conditions. The mid-log phase cultures were washed and placed in fresh media and adjusted to an OD of 0.2. Compounds were then administered at 2× MIC for 4 hours. After 4 hours, the bacterial cells were harvested and resuspended in trizol. RNA extraction was performed as described below in the “Bacterial RNA extraction” section.

### Bacterial RNA extraction

Trizol samples containing bacterial cell lysates were placed in a bead beater for three cycles of 45 s each, resting on ice for 2 min between cycles. Following bead beating, chloroform was added, gently mixed with the lysates, and separation via centrifugation was performed. The aqueous phase was added to an equal volume of pure ethanol, and RNA was purified using a Qiagen RNeasy Kit followed by the Turbo DNase Kit to degrade DNA. Library prep and sequencing were performed by SeqCenter.

### RNA-seq analyses

Htseq-counts were input into the DeSeq2 pipeline in R for differential expression analyses. Counts were pre-filtered to eliminate low read counts (<10 counts/read), normalized based on gene length, and batch effects were removed via limma. Genes considered to be differentially expressed need to have both a *P*-value of ≤0.05 and a log_2_fc of >1 (upregulated) or <−1 (downregulated). Functional analyses were performed using STRING-db.org.

## SUPPLEMENTAL MATERIAL

Supplemental material A aac.00369-25-s0001.docx Fig. S1 to S14; Tables S1 to S8.

Supplemental material B aac.00369-25-s0002.xlsx Mutation analysis between wild-type *M. tuberculosis* and speirobactin-resistant mutants.

Supplemental material C aac.00369-25-s0003.xlsx Raw transcriptomic data of *M. tuberculosis* treated with speirobactin, ciprofloxacin, moxifloxacin, ethambutol, and isoniazid.

Supplemental material D aac.00369-25-s0004.xlsx Genes from *M. tuberculosis* that are upregulated and downregulated when treated with speirobactin, ciprofloxacin, moxifloxacin, ethambutol, and isoniazid.

ASM does not own the copyrights to Supplemental Material that may be linked to, or accessed through, an article. The authors have granted ASM a non-exclusive, world-wide license to publish the Supplemental Material files. Please contact the corresponding author directly for reuse.
