# Supplementary material for: A chlorinated diketopiperazine antibiotic targets Mycobacterium tuberculosis
Source: Antimicrob Agents Chemother. 2025 Jul 31;69(9):e00369-25. doi: 10.1128/aac.00369-25 (PMC12406682; doi:10.1128/aac.00369-25)
Supplement: Supplemental material A — Fig. S1 to S14; Tables S1 to S8. [file aac.00369-25-s0001.docx]

**A Chlorinated Diketopiperazine Antibiotic Targets *Mycobacterium Tuberculosis***

Libang Liang,^†, §^ Jeffery Quigley,^†, §^ Monique Theriault, ^†^ Akira Iinishi,^†^ Rachel Bargabos,^†^ Madeleine Morrissette,^†^ Megan Ghiglieri,^†^ Tom Curtis,^†^ Rachel Corsetti,^†^ Sangkeun Son,^†^ Bishwarup Sarkar,^†^ and Kim Lewis^*,†^

^†^Antimicrobial Discovery Center, Department of Biology, Northeastern University, 360 Huntington Ave., Boston, MA, 02115, USA

^*^Corresponding author

^§^These authors contributed equally

**Table of Content**

Fig. S1. ^1^H NMR (500 MHz) of speirobactin in DMSO-*d_6_*.

Fig. S2. ^13^C NMR (175 MHz) of speirobactin in DMSO-*d_6_*.

Fig. S3. COSY NMR (700 MHz) of speirobactin in DMSO-*d_6_*.

Fig. S4. TOCSY NMR (700 MHz) of speirobactin in DMSO-*d_6_*.

Fig. S5. NOESY NMR (700 MHz) of speirobactin in DMSO-*d_6_*.

Fig. S6. ^1^H-^13^C HSQC NMR (700 / 175 MHz) of speirobactin in DMSO-*d_6_*

Fig. S7. ^1^H-^13^C HMBC NMR (700 / 175 MHz) of speirobactin in DMSO-*d_6_*.

Fig. S8. Mass spectrometry analysis of speirobactin.

Fig. S9. HPLC separation of speirobactin enantiomers using a COSMOSIL CHiRAL Series 5C HPLC column.

Fig. S10. Marfey’s analysis of speirobactin by LC-HRMS.

Fig. S11. UV absorbance of speirobactin.

Fig. S12. Sequences of oligonucleotides used to make targeted mutations by single stranded recombineering in Mtb.

Fig. S13. STRING plot of upregulated Genes induced only by speirobactin, but not by ciprofloxacin and moxifloxacin from RNA-seq studies (log2fc >1, p-value ≤0.05).

Fig. S14. STRING plot of downregulated Genes induced only by speirobactin, but not by ciprofloxacin and moxifloxacin from RNA-seq studies (log2fc >1, p-value ≤0.05).

Table S1. The *Photorhabdus* strains used for antitubercular screening.

Table S2. Combination of strains and fermentation media that produced antitubercular compounds (>90% inhibition) using non-concentrated supernatants.

Table S3. Media in the screening that induced production for antitubercular compounds in *Photorhabdus* (concentration g/L).

Table S4. 1H / 13C NMR (700 / 175 MHz) chemical shifts of speirobactin in DMSO-*d_6_*.

Table S5*.* Top 100 BLASTp hits from NCBI with query protein sequence of gene E.

Table S6. Mutations from speirobactin-resistant *M. tuberculosis* mutants.

Table S7. Upregulated Genes shared by speirobactin, ciprofloxacin and moxifloxacin from RNA-seq studies (log2fc >1, p-value ≤0.05).

Table S8. Downregulated Genes shared by speirobactin, ciprofloxacin and moxifloxacin from RNA-seq studies (log2fc >1, p-value ≤0.05).

*
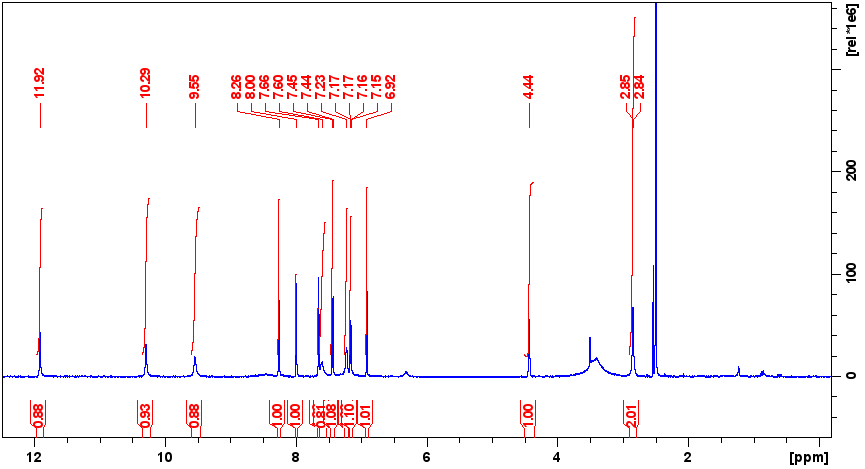
*

Fig. S1. ^1^H NMR (500 MHz) of speirobactin in DMSO-*d_6_*.


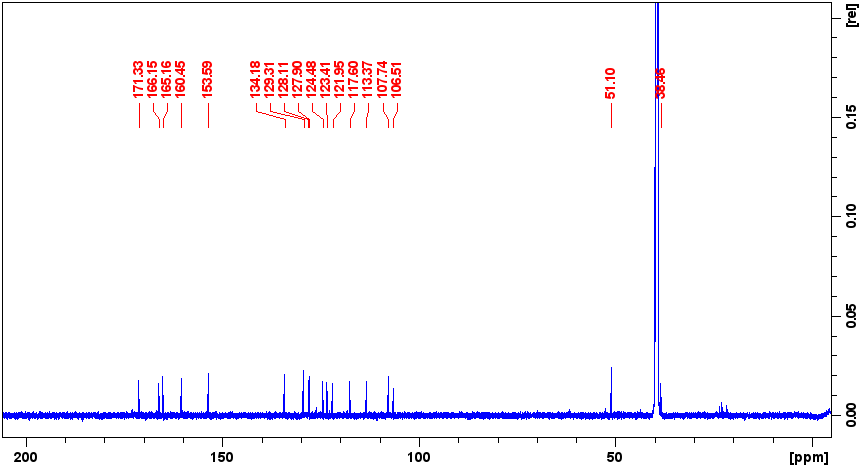


Fig. S2. ^13^C NMR (175 MHz) of speirobactin in DMSO-*d_6_*.


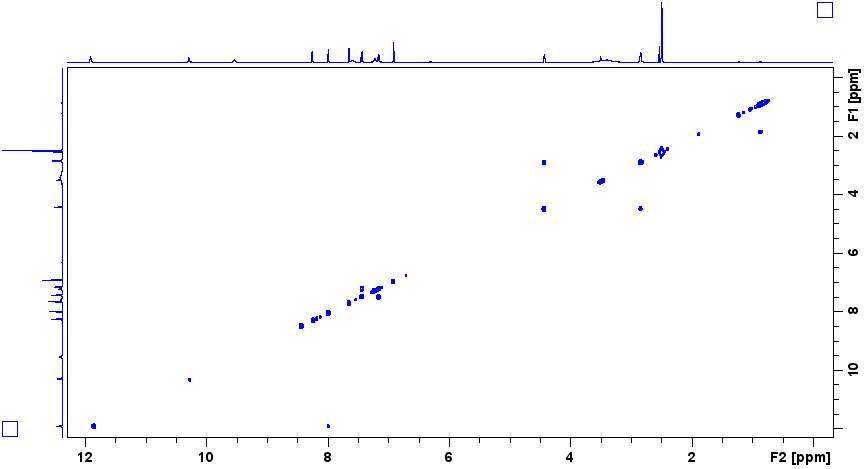


Fig. S3. COSY NMR (700 MHz) of speirobactin in DMSO-*d_6_*.


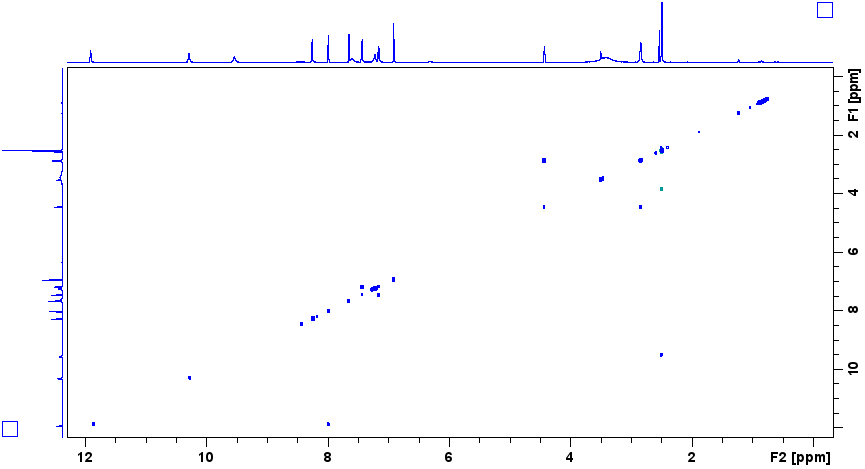


Fig. S4. TOCSY NMR (700 MHz) of speirobactin in DMSO-*d_6_*.


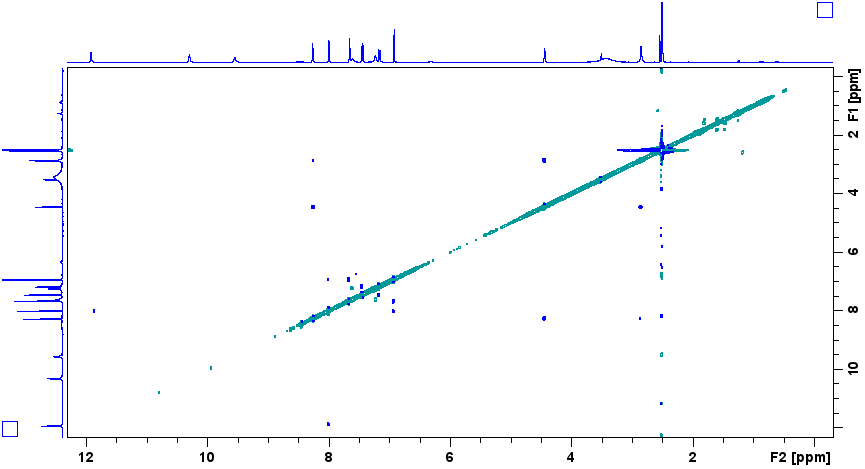


Fig. S5. NOESY NMR (700 MHz) of speirobactin in DMSO-*d_6_*.


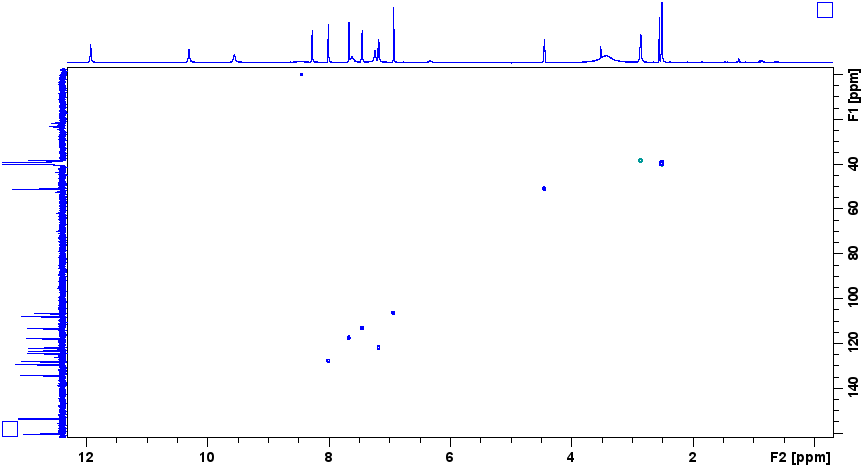


Fig. S6. ^1^H-^13^C HSQC NMR (700 / 175 MHz) of speirobactin in DMSO-*d_6_*.


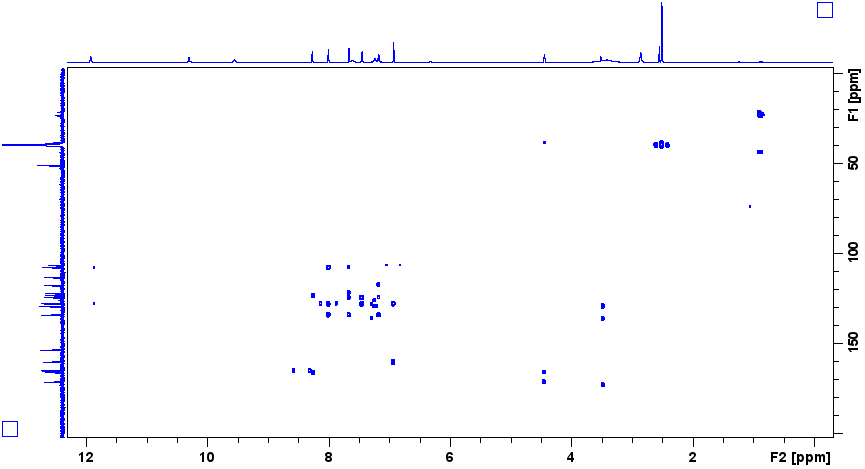


Fig. S7. ^1^H-^13^C HMBC NMR (700 / 175 MHz) of speirobactin in DMSO-*d_6_*.


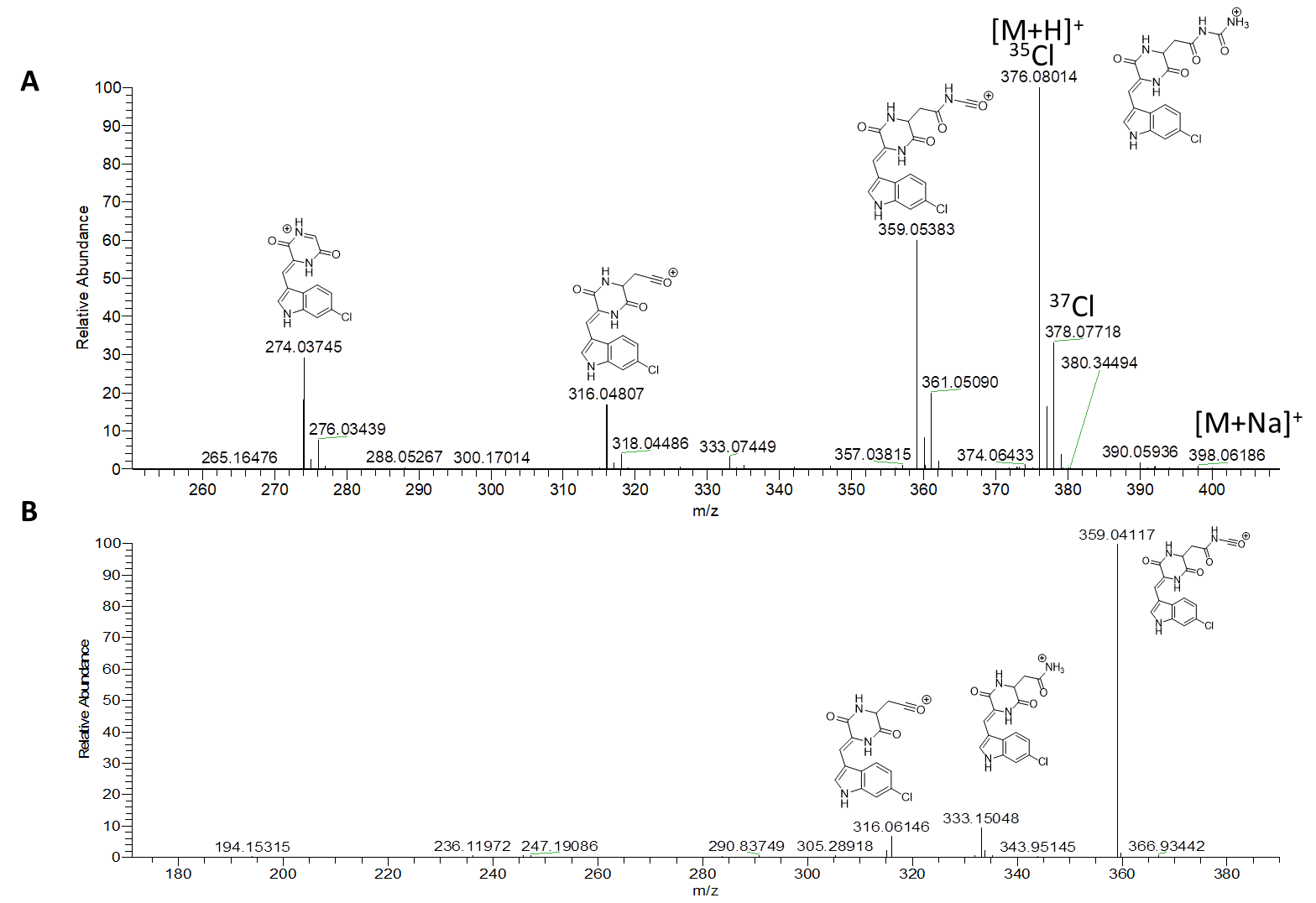


Fig. S8. Mass spectrometry analysis of speirobactin. (A) Thermo LTQ Velos LC-MS mode. (B) Thermo LTQ Velos MS^2^ fragmentation pattern by selecting the parent ion with an *m/z* value at 376.08 and fragmenting 28.00 eV.


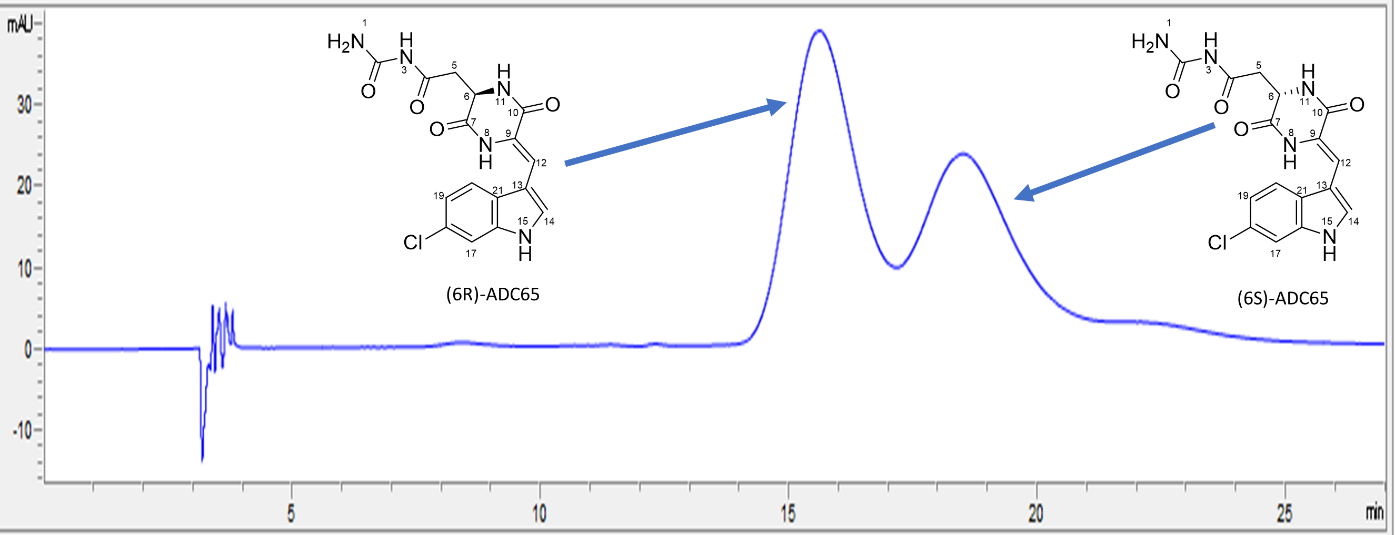


Fig. S9. HPLC separation of speirobactin enantiomers using a COSMOSIL CHiRAL Series 5C HPLC column (4.6 mm × 250 mm, Log # 15790-71). Isocratic mobile phase consisting of 20% ACN (0.1% formic acid) and 80% H2O (0.1% formic acid) was used at 1 mL/min. UV detector monitored at 342 nm.


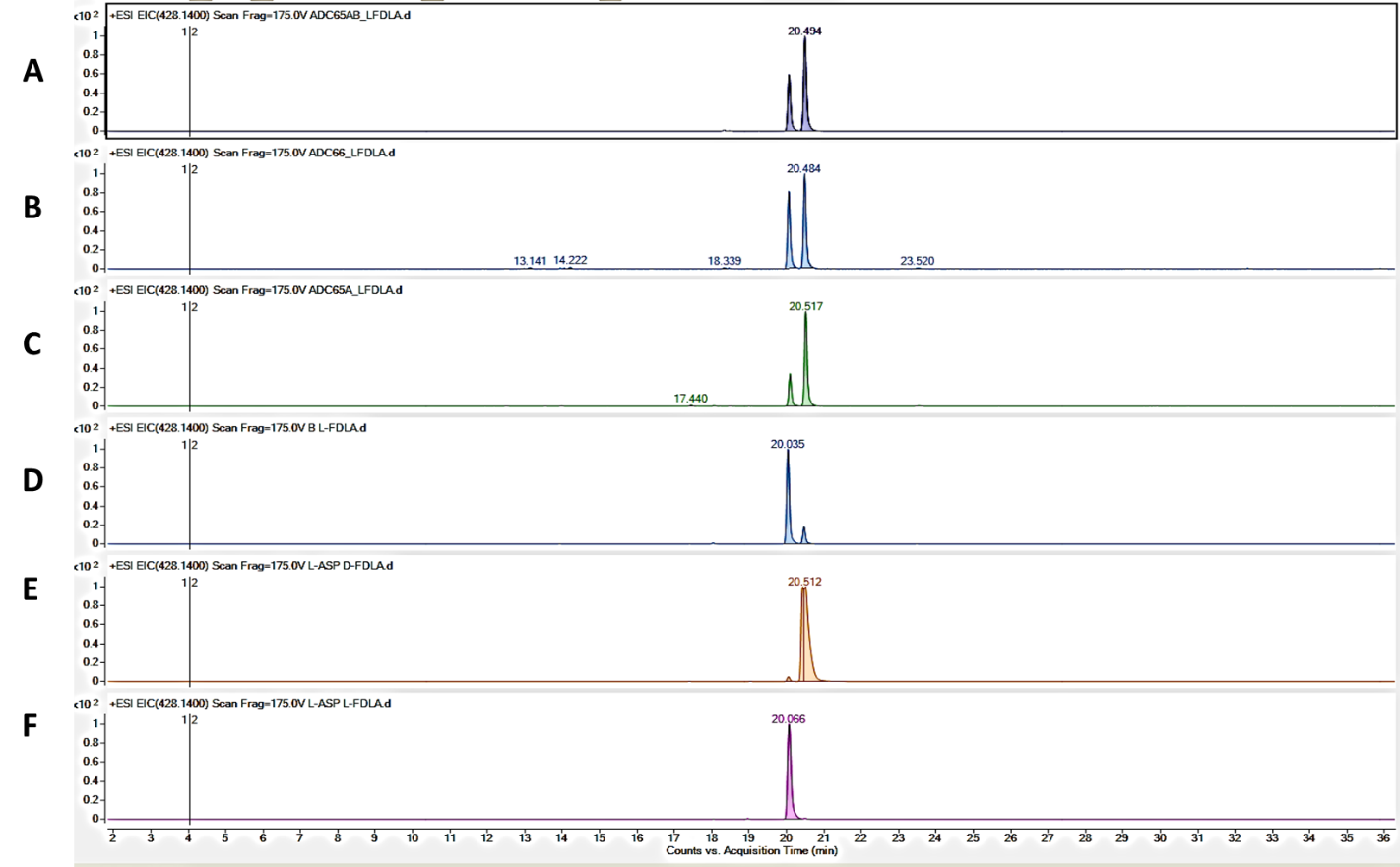


Fig. S10. Marfey’s analysis of speirobactin by LC-HRMS. (A) speirobactin reacted with L-FDLA; (B) An isolated analog of speirobactin reacted with L-FDLA, the analog has an [M+H]^+^ 333.07 and its isotopic peak 335.07 at about a third of its intensity ; (C) (6R)-speirobactin reacted with L-FDLA; (D) (6S)-speirobactin reacted with L-FDLA; (E) L-ASP reacted with D-FDLA; (F) L-ASP reacted with L-FDLA.


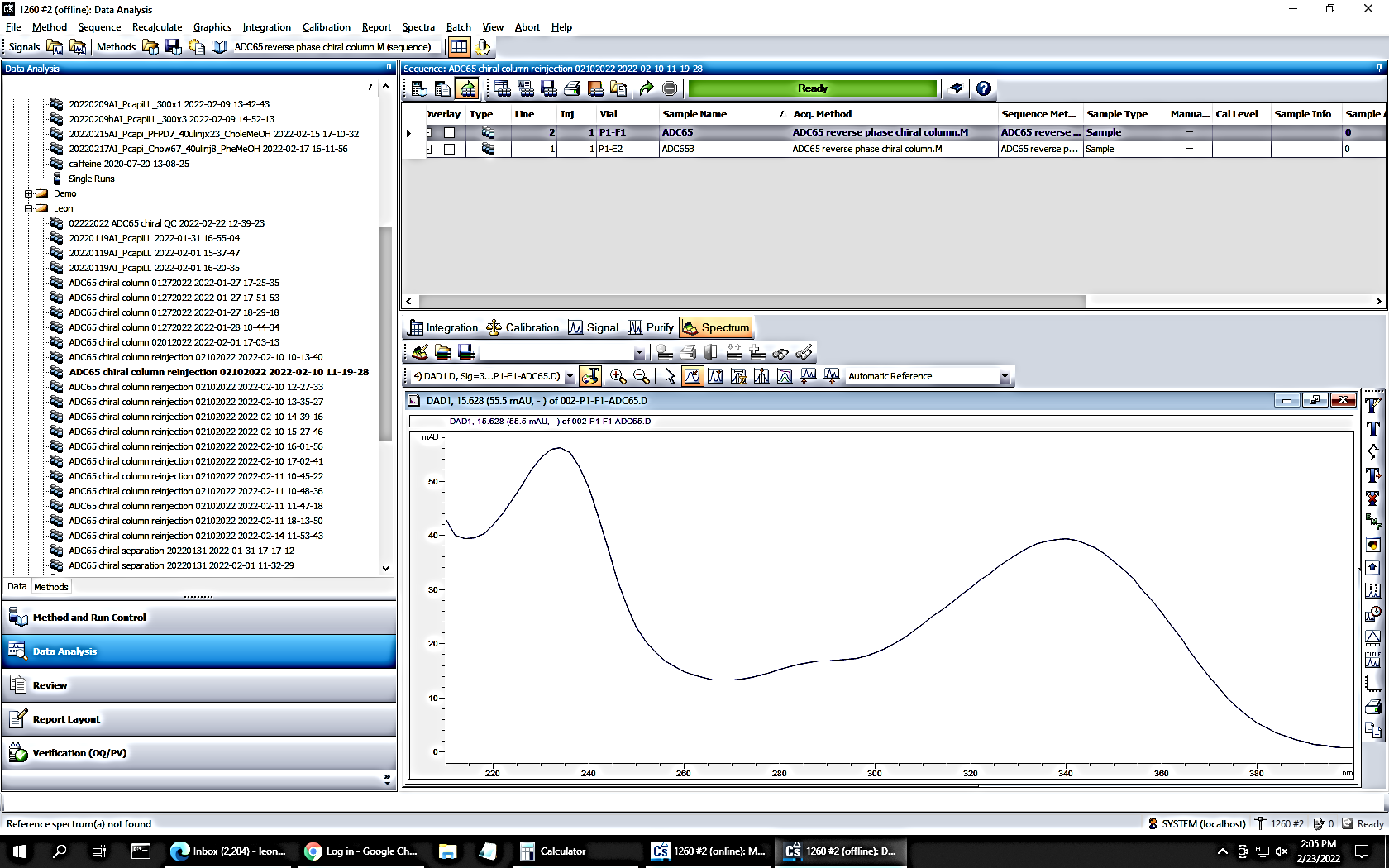


Fig. S11. UV absorbance of speirobactin from an Agilent HPLC UV detector in 25% ACN (0.1% formic acid), 75% H_2_O (0.1% formic acid).

*gyrA* D89G

5’-GGGCCATGCGCACCAGGCTGTCGTAGATCGACGCGCCGCCGTGCGGGTGGTAGTTGCCCATGGTCTCGGC-3’

*gyrA* Q101H

5’- GGCCGTCCACCAGCGGGTAGCGCAGCGACCAGGGGTGGGCCATGCGCACCAGGCTGTCGTAGATCGACGC-3’

*gyrA* R128K

5’- CCATCGCCAACGGGGTCAGCCGGGCTTCGGTGTACTTCATCGCCGCCGGTGGGTCATTGCCTGGCGAGCC-3’

Fig. S12. Sequences of oligonucleotides used to make targeted mutations by single stranded recombineering in Mtb.


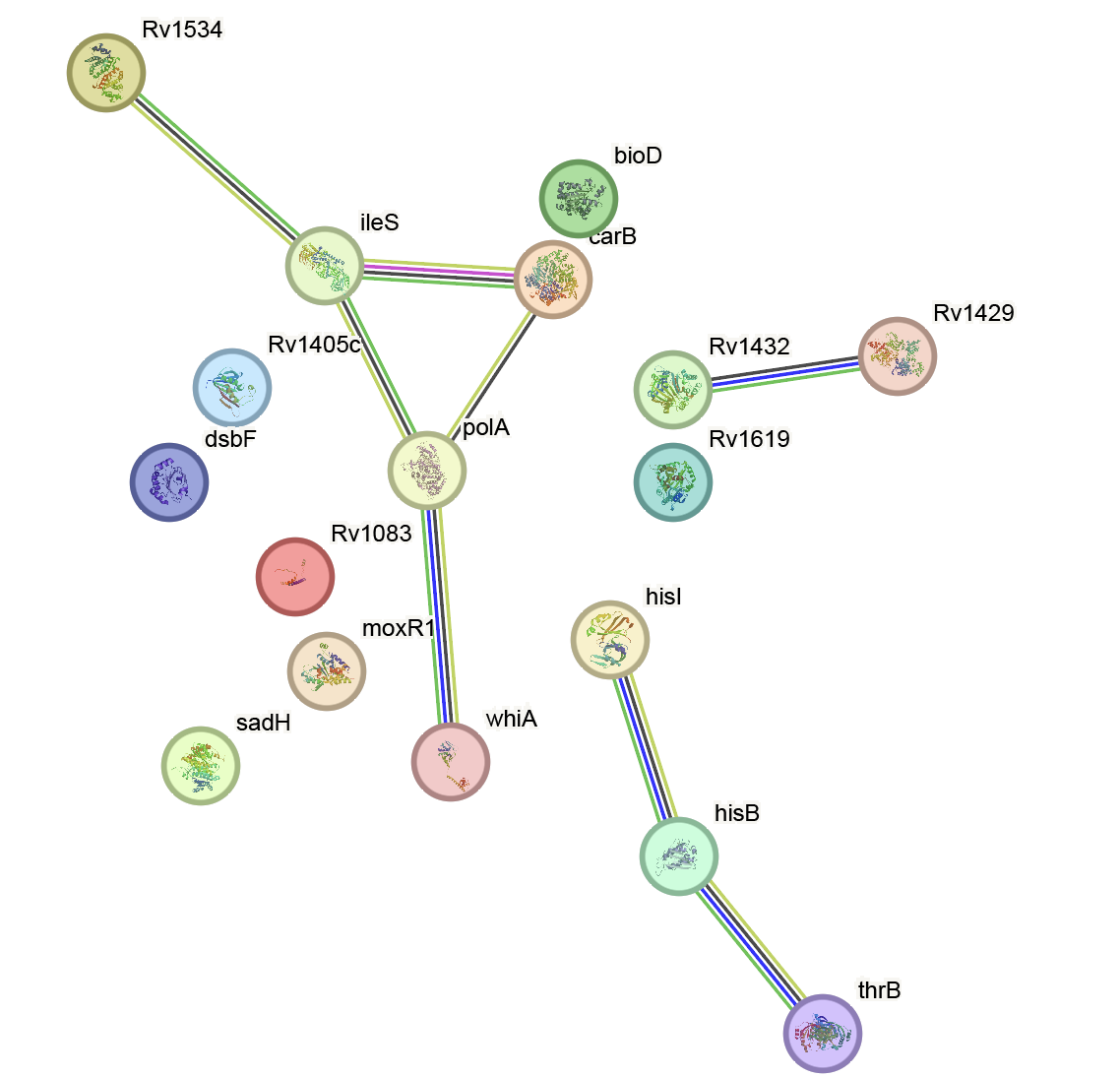

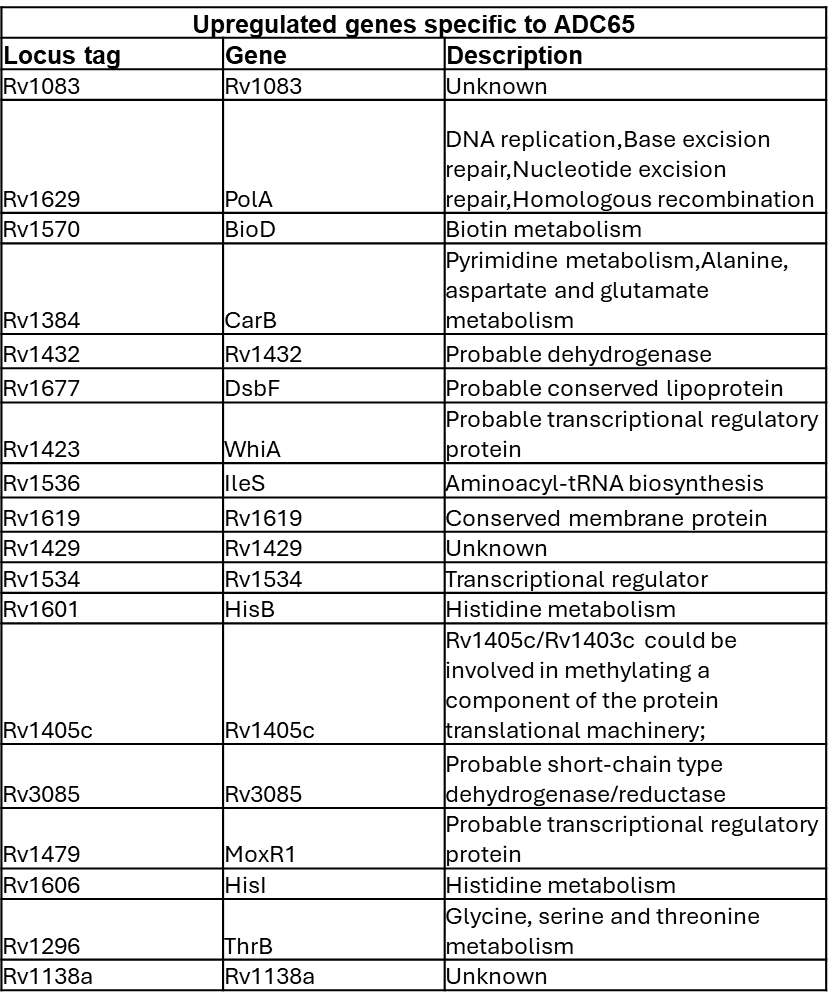


Fig. S13. STRING plot of upregulated genes induced only by speirobactin, but not by ciprofloxacin and moxifloxacin from RNA-seq studies (log2fc >1, p-value ≤0.05).


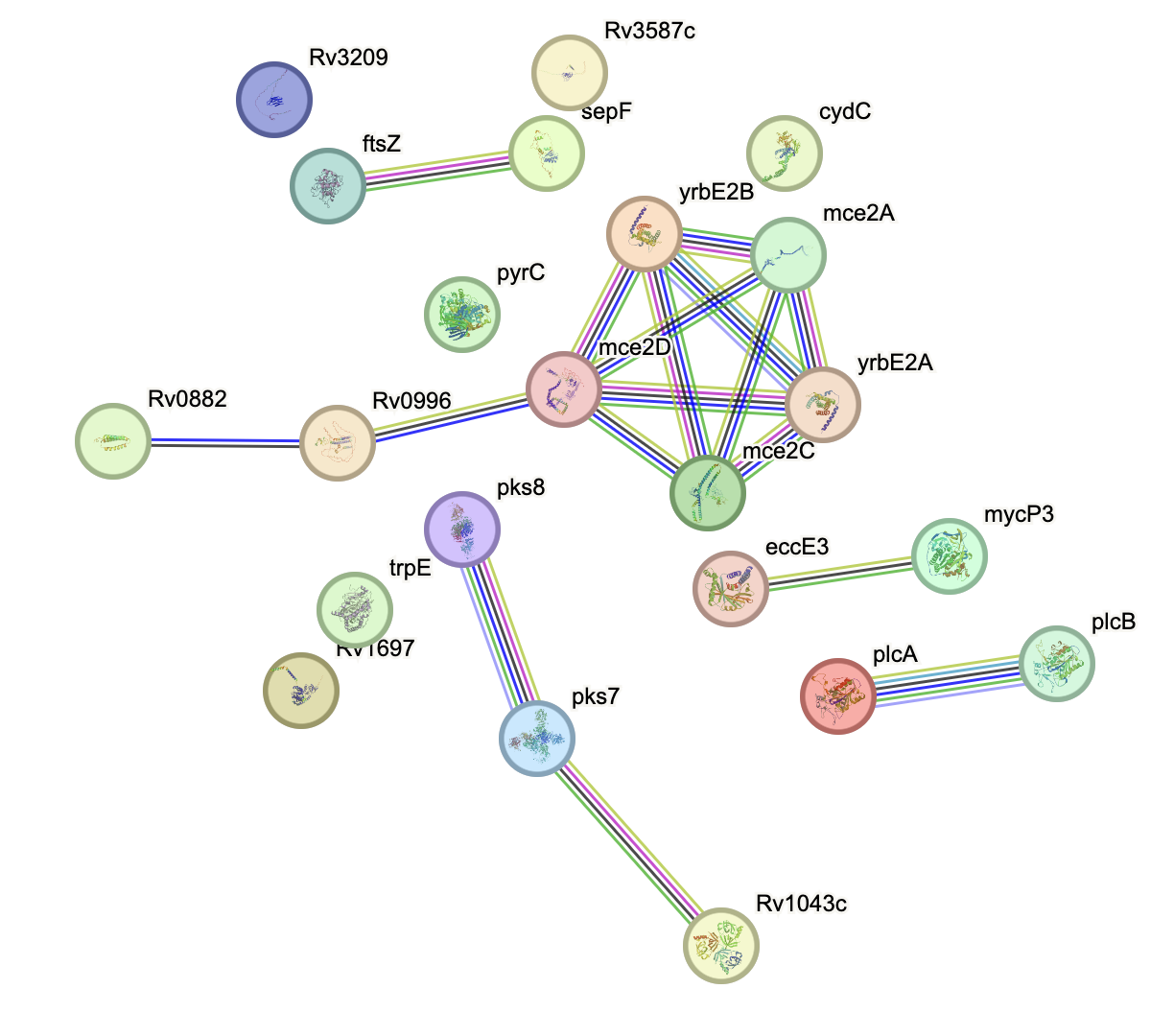

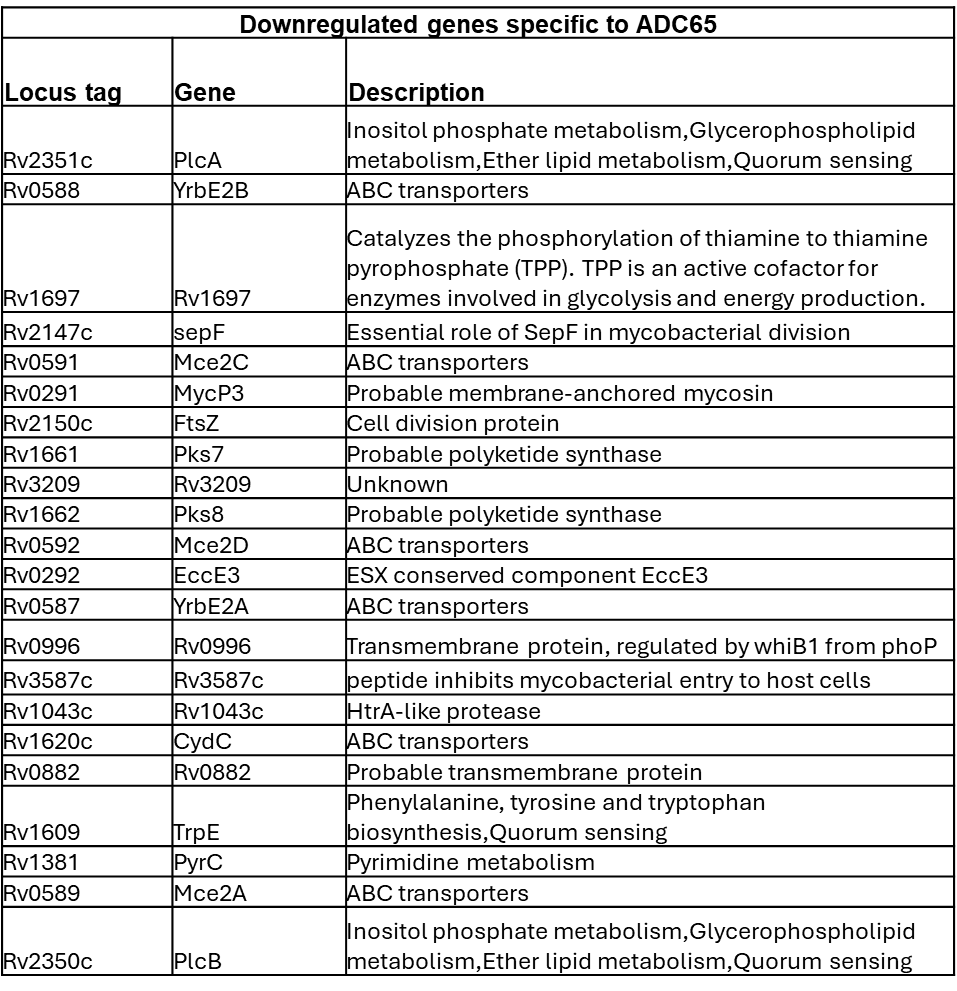


Fig. S14. STRING plot of downregulated genes induced only by speirobactin, but not by ciprofloxacin and moxifloxacin from RNA-seq studies (log2fc >1, p-value ≤0.05).

Table S1. The *Photorhabdus* strains used for antitubercular screening.

| **KLE_ID*** | **Genus** | **species_ID** | **Subspecies** | **Strain** |
| --- | --- | --- | --- | --- |
| KLE10356 | *Photorhabdus* | *temperata* |  | Wx9Hyper(P48) |
| KLE10359 | *Photorhabdus* | *temperata* |  | Hepialius |
| KLE10360 | *Photorhabdus* | *temperata* |  | OH1 |
| KLE10363 | *Photorhabdus* | *temperata* |  | Pmeg |
| KLE10364 | *Photorhabdus* | *temperata* |  | He86 |
| KLE10365 | *Photorhabdus* | *temperata* | *temperata* | HSH2 |
| KLE10366 | *Photorhabdus* | *temperata* | *temperata* | K122 |
| KLE10369 | *Photorhabdus* | *luminescens* | *luminescens* | Hm |
| KLE10370 | *Photorhabdus* | *luminescens* | *luminescens* | Hb |
| KLE11397 | *Photorhabdus* | *temperata* | *temperata* |  |
| KLE11398 | *Photorhabdus* | *temperata* | *temperata* |  |
| KLE11400 | *Photorhabdus* | *temperata* | *temperata* |  |
| KLE11401 | *Photorhabdus* | *luminescens* | *luminescens* |  |
| KLE11403 | *Photorhabdus* | *temperata* | *temperata* |  |
| KLE11405 | *Photorhabdus* | *luminescens* | *luminescens* |  |
| KLE11406 | *Photorhabdus* | *temperata* | *temperata* |  |
| KLE11408 | *Photorhabdus* | *luminescens* | *luminescens* |  |

* KLE numbers are strain IDs in Kim Lewis lab at Northeastern University.

Table S2. Combination of strains and fermentation media that produced antitubercular compounds (>90% inhibition) using non-concentrated supernatants.

| **KLE_ID** | **Genus** | **species_ID** | **Subspecies** | **Strain** | **Fermentation media** |
| --- | --- | --- | --- | --- | --- |
| KLE10364 | *Photorhabdus* | *temperata* |  | He86 | M5, M15, LB, TSB |
| KLE10366 | *Photorhabdus* | *temperata* | *temperata* | K122 | M4, LB |
| KLE11398 | *Photorhabdus* | *temperata* | *temperata* |  | M10, M11 |

Table S3. Media in the screening that induced production for antitubercular compounds in *Photorhabdus* (concentration g/L).

M5:

| Glucose | 40 |
| --- | --- |
| Dried yeast | 5 |
| K_2_HPO_4_ | 1 |
| NaCl | 1 |
| (NH_4_)SO_4_ | 2 |
| CaCO_3_ | 2 |

M15:

| Bacto Peptone | 10 |
| --- | --- |
| K_2_HPO_4_ | 1 |
| MgSO_4_, 7H_2_O | 1 |
| (NH_4_)_2_SO_4_ | 2 |
| CaCO_3_ | 2 |
| NaCl | 10 |

M4:

| Yeast Extract | 5 |
| --- | --- |
| Tryptone | 5 |
| MgSO_4_-7H_2_0 | 5 |
| NaCl | 30 |

M10:

| Glucose | 10 |
| --- | --- |
| Starch | 10 |
| Soybean meal | 15 |
| K_2_HPO_4_ | 1 |
| NaCl | 3 |
| MgSO_4_-7H_2_0 | 1 |

M11:

| glucose | 0.4 |
| --- | --- |
| galactose | 0.8 |
| maltose | 0.8 |
| dextrin | 1.6 |
| Bacto Soytone | 0.8 |
| ammonium sulfate | 0.3 |

TSB:

| casein peptone (pancreatic) | 17 |
| --- | --- |
| dipotassium hydrogen phosphate | 2.5 |
| glucose | 2.5 |
| sodium chloride | 5 |
| soya peptone (papain digest.) | 3 |

LB:

| NaCl | 10 |
| --- | --- |
| Tryptone | 10 |
| Yeast Extract | 5 |

Table S4. 1H / 13C NMR (700 / 175 MHz) chemical shifts of speirobactin in DMSO-*d_6_*.

| Position | δC, type | δH, mult (J, Hz) | COSY | ^1^H-^13^C HMBC |
| --- | --- | --- | --- | --- |
| 1 | NH_2_ | 7.60/7.23, broad | - | - |
| 2 | 153.6, C | - | - | - |
| 3 | NH | 10.29, s | - | - |
| 4 | 171.3, C | - | - | - |
| 5 | 38.5, CH_2_ | 2.85, d (5.0) | 6 | 6, 7, 4 |
| 6 | 51.1, CH | 4.44, dt (1.5, 5.0) | 5, 11 | 5, 10, 7, 4 |
| 7 | 166.2, C | - | - | - |
| 8 | NH | 9.55, s | - | - |
| 9 | 123.4, C | - | - | - |
| 10 | 160.5, C | - | - | - |
| 11 | NH | 8.26, s | - | 6, 12, 10, 7, 9 |
| 12 | 106.5, CH | 6.92, s | - | 9, 7, 10, 14 |
| 13 | 107.7, C | - | - | - |
| 14 | 127.9, CH | 8.00, s | - | 21, 16, 13 |
| 15 | NH | 11.92, s | - | 13, 16, 21 |
| 16 | 134.2, C | - | - | - |
| 17 | 117.6, CH | 7.65, d (2.0) | - | 13, 20, 19, 18, 16, 21 |
| 18 | 124.5, C | - | - | - |
| 19 | 122.0, CH | 7.16, dd (8.6, 2.0) | 20 | 17, 18, 16 |
| 20 | 113.4, CH | 7.44, d (8.6) | 19 | 17, 18, 21 |
| 21 | 128.1, C | - | - | - |

Table S5*.* Top 100 BLASTp hits from NCBI with query protein sequence of gene E.

| Description | Coverage | Identity% | Accession No. | E value |
| --- | --- | --- | --- | --- |
| pentapeptide repeat-containing protein [Photorhabdus tasmaniensis] | 100% | 100 | [WP_133816294.1](https://www.ncbi.nlm.nih.gov/protein/WP_133816294.1?report=genbank&log$=prottop&blast_rank=1&RID=1HY1HUD3013) | 3.00E-174 |
| pentapeptide repeat-containing protein [Photorhabdus thracensis] | 100% | 95.62 | [WP_021324659.1](https://www.ncbi.nlm.nih.gov/protein/WP_021324659.1?report=genbank&log$=prottop&blast_rank=2&RID=1HY1HUD3013) | 6.00E-167 |
| hypothetical protein VY86_05170 [Photorhabdus thracensis] | 100% | 95.62 | [AKH62813.1](https://www.ncbi.nlm.nih.gov/protein/AKH62813.1?report=genbank&log$=prottop&blast_rank=3&RID=1HY1HUD3013) | 4.00E-166 |
| pentapeptide repeat-containing protein [Photorhabdus sp. CRCIA-P01] | 100% | 88.05 | [WP_198672851.1](https://www.ncbi.nlm.nih.gov/protein/WP_198672851.1?report=genbank&log$=prottop&blast_rank=4&RID=1HY1HUD3013) | 1.00E-151 |
| pentapeptide repeat-containing protein [Photorhabdus kayaii] | 100% | 87.25 | [NDL14150.1](https://www.ncbi.nlm.nih.gov/protein/NDL14150.1?report=genbank&log$=prottop&blast_rank=5&RID=1HY1HUD3013) | 2.00E-150 |
| pentapeptide repeat-containing protein [Photorhabdus luminescens] | 100% | 86.85 | [PQQ37750.1](https://www.ncbi.nlm.nih.gov/protein/PQQ37750.1?report=genbank&log$=prottop&blast_rank=6&RID=1HY1HUD3013) | 3.00E-150 |
| pentapeptide repeat-containing protein [Photorhabdus laumondii] | 100% | 86.85 | [WP_206606980.1](https://www.ncbi.nlm.nih.gov/protein/WP_206606980.1?report=genbank&log$=prottop&blast_rank=7&RID=1HY1HUD3013) | 3.00E-150 |
| pentapeptide repeat-containing protein [Photorhabdus] | 100% | 87.25 | [WP_205068580.1](https://www.ncbi.nlm.nih.gov/protein/WP_205068580.1?report=genbank&log$=prottop&blast_rank=8&RID=1HY1HUD3013) | 4.00E-150 |
| pentapeptide repeat-containing protein [Photorhabdus luminescens] | 99% | 86 | [WP_088373606.1](https://www.ncbi.nlm.nih.gov/protein/WP_088373606.1?report=genbank&log$=prottop&blast_rank=9&RID=1HY1HUD3013) | 4.00E-148 |
| pentapeptide repeat-containing protein [Photorhabdus stackebrandtii] | 100% | 84.86 | [WP_166285204.1](https://www.ncbi.nlm.nih.gov/protein/WP_166285204.1?report=genbank&log$=prottop&blast_rank=10&RID=1HY1HUD3013) | 2.00E-147 |
| pentapeptide repeat-containing protein [Photorhabdus luminescens subsp. sonorensis] | 99% | 85.6 | [TNH43620.1](https://www.ncbi.nlm.nih.gov/protein/TNH43620.1?report=genbank&log$=prottop&blast_rank=11&RID=1HY1HUD3013) | 1.00E-146 |
| pentapeptide repeat-containing protein [Photorhabdus luminescens] | 99% | 85.6 | [WP_218117394.1](https://www.ncbi.nlm.nih.gov/protein/WP_218117394.1?report=genbank&log$=prottop&blast_rank=12&RID=1HY1HUD3013) | 2.00E-146 |
| pentapeptide repeat-containing protein [Photorhabdus khanii] | 100% | 83.67 | [WP_132355803.1](https://www.ncbi.nlm.nih.gov/protein/WP_132355803.1?report=genbank&log$=prottop&blast_rank=13&RID=1HY1HUD3013) | 3.00E-145 |
| pentapeptide repeat-containing protein [Photorhabdus] | 100% | 84.06 | [WP_051477206.1](https://www.ncbi.nlm.nih.gov/protein/WP_051477206.1?report=genbank&log$=prottop&blast_rank=14&RID=1HY1HUD3013) | 4.00E-144 |
| pentapeptide repeat-containing protein [Xenorhabdus szentirmaii] | 100% | 82.47 | [WP_099138954.1](https://www.ncbi.nlm.nih.gov/protein/WP_099138954.1?report=genbank&log$=prottop&blast_rank=15&RID=1HY1HUD3013) | 2.00E-141 |
| pentapeptide repeat-containing protein [Xenorhabdus sp. CUL] | 100% | 82.07 | [MBD2791917.1](https://www.ncbi.nlm.nih.gov/protein/MBD2791917.1?report=genbank&log$=prottop&blast_rank=16&RID=1HY1HUD3013) | 3.00E-141 |
| pentapeptide repeat-containing protein [Photorhabdus heterorhabditis] | 100% | 82.87 | [WP_054480763.1](https://www.ncbi.nlm.nih.gov/protein/WP_054480763.1?report=genbank&log$=prottop&blast_rank=17&RID=1HY1HUD3013) | 9.00E-141 |
| pentapeptide repeat-containing protein [Xenorhabdus szentirmaii] | 100% | 82.07 | [WP_051462493.1](https://www.ncbi.nlm.nih.gov/protein/WP_051462493.1?report=genbank&log$=prottop&blast_rank=18&RID=1HY1HUD3013) | 1.00E-140 |
| pentapeptide repeat-containing protein [Xenorhabdus sp. ZM] | 100% | 82.07 | [MBD2803423.1](https://www.ncbi.nlm.nih.gov/protein/MBD2803423.1?report=genbank&log$=prottop&blast_rank=19&RID=1HY1HUD3013) | 2.00E-140 |
| pentapeptide repeat-containing protein [Endozoicomonas sp. OPT23] | 98% | 62.9 | [WP_153767566.1](https://www.ncbi.nlm.nih.gov/protein/WP_153767566.1?report=genbank&log$=prottop&blast_rank=20&RID=1HY1HUD3013) | 3.00E-105 |
| pentapeptide repeat-containing protein [Marinomonas sp. MED121] | 98% | 62.1 | [WP_009831720.1](https://www.ncbi.nlm.nih.gov/protein/WP_009831720.1?report=genbank&log$=prottop&blast_rank=21&RID=1HY1HUD3013) | 6.00E-103 |
| pentapeptide repeat-containing protein [Desulfobacterium sp.] | 94% | 64.71 | [MBI9092654.1](https://www.ncbi.nlm.nih.gov/protein/MBI9092654.1?report=genbank&log$=prottop&blast_rank=22&RID=1HY1HUD3013) | 2.00E-102 |
| pentapeptide repeat-containing protein [Crocinitomicaceae bacterium] | 99% | 61.6 | [MBL4862598.1](https://www.ncbi.nlm.nih.gov/protein/MBL4862598.1?report=genbank&log$=prottop&blast_rank=23&RID=1HY1HUD3013) | 1.00E-99 |
| pentapeptide repeat-containing protein [Chloroflexi bacterium] | 98% | 59.68 | [NQT71730.1](https://www.ncbi.nlm.nih.gov/protein/NQT71730.1?report=genbank&log$=prottop&blast_rank=24&RID=1HY1HUD3013) | 4.00E-96 |
| hypothetical protein AHAT_20970 [Agarivorans sp. Toyoura001] | 99% | 55.02 | [GDY26207.1](https://www.ncbi.nlm.nih.gov/protein/GDY26207.1?report=genbank&log$=prottop&blast_rank=25&RID=1HY1HUD3013) | 2.00E-90 |
| pentapeptide repeat-containing protein [Agarivorans sp. Toyoura001] | 99% | 55.02 | [WP_235836203.1](https://www.ncbi.nlm.nih.gov/protein/WP_235836203.1?report=genbank&log$=prottop&blast_rank=26&RID=1HY1HUD3013) | 3.00E-90 |
| pentapeptide repeat-containing protein [Agarivorans sp. B2Z047] | 99% | 54.62 | [WP_152783222.1](https://www.ncbi.nlm.nih.gov/protein/WP_152783222.1?report=genbank&log$=prottop&blast_rank=27&RID=1HY1HUD3013) | 6.00E-89 |
| pentapeptide repeat-containing protein [Agarivorans albus] | 99% | 53.82 | [WP_016402349.1](https://www.ncbi.nlm.nih.gov/protein/WP_016402349.1?report=genbank&log$=prottop&blast_rank=28&RID=1HY1HUD3013) | 6.00E-88 |
| pentapeptide repeat-containing protein [Gammaproteobacteria bacterium] | 98% | 52.85 | [MBA2654007.1](https://www.ncbi.nlm.nih.gov/protein/MBA2654007.1?report=genbank&log$=prottop&blast_rank=29&RID=1HY1HUD3013) | 1.00E-77 |
| pentapeptide repeat-containing protein [Fastidiosibacter lacustris] | 99% | 50.6 | [WP_116964694.1](https://www.ncbi.nlm.nih.gov/protein/WP_116964694.1?report=genbank&log$=prottop&blast_rank=30&RID=1HY1HUD3013) | 3.00E-73 |
| pentapeptide repeat-containing protein [Fangia hongkongensis] | 95% | 46.86 | [WP_018300761.1](https://www.ncbi.nlm.nih.gov/protein/WP_018300761.1?report=genbank&log$=prottop&blast_rank=31&RID=1HY1HUD3013) | 5.00E-65 |
| pentapeptide repeat-containing protein [Pseudomonas mucoides] | 92% | 43.35 | [WP_194940342.1](https://www.ncbi.nlm.nih.gov/protein/WP_194940342.1?report=genbank&log$=prottop&blast_rank=32&RID=1HY1HUD3013) | 1.00E-61 |
| pentapeptide repeat-containing protein [Pseudomonas sp. URMO17WK12:I11] | 92% | 43.35 | [WP_059182601.1](https://www.ncbi.nlm.nih.gov/protein/WP_059182601.1?report=genbank&log$=prottop&blast_rank=33&RID=1HY1HUD3013) | 2.00E-61 |
| pentapeptide repeat-containing protein [Pseudomonas lini] | 93% | 44.44 | [WP_222839683.1](https://www.ncbi.nlm.nih.gov/protein/WP_222839683.1?report=genbank&log$=prottop&blast_rank=34&RID=1HY1HUD3013) | 1.00E-60 |
| pentapeptide repeat-containing protein [Pseudomonas sp. BF-R-26] | 93% | 44.44 | [WP_223570887.1](https://www.ncbi.nlm.nih.gov/protein/WP_223570887.1?report=genbank&log$=prottop&blast_rank=35&RID=1HY1HUD3013) | 2.00E-60 |
| pentapeptide repeat-containing protein [Pseudomonas sp. MG-9] | 95% | 42.08 | [WP_215500087.1](https://www.ncbi.nlm.nih.gov/protein/WP_215500087.1?report=genbank&log$=prottop&blast_rank=36&RID=1HY1HUD3013) | 1.00E-59 |
| pentapeptide repeat-containing protein [unclassified Pseudomonas] | 93% | 42.74 | [WP_178113332.1](https://www.ncbi.nlm.nih.gov/protein/WP_178113332.1?report=genbank&log$=prottop&blast_rank=37&RID=1HY1HUD3013) | 4.00E-59 |
| fluoroquinolone resistance protein [Pseudomonas sp. SJZ083] | 93% | 42.74 | [TWC11588.1](https://www.ncbi.nlm.nih.gov/protein/TWC11588.1?report=genbank&log$=prottop&blast_rank=38&RID=1HY1HUD3013) | 6.00E-59 |
| pentapeptide repeat-containing protein [Agarivorans aestuarii] | 99% | 39.36 | [WP_221075068.1](https://www.ncbi.nlm.nih.gov/protein/WP_221075068.1?report=genbank&log$=prottop&blast_rank=39&RID=1HY1HUD3013) | 2.00E-53 |
| pentapeptide repeat-containing protein [Kistimonas asteriae] | 94% | 37.71 | [WP_211830403.1](https://www.ncbi.nlm.nih.gov/protein/WP_211830403.1?report=genbank&log$=prottop&blast_rank=40&RID=1HY1HUD3013) | 3.00E-51 |
| pentapeptide repeat-containing protein [Oceanospirillaceae bacterium] | 98% | 37.9 | [NRA20515.1](https://www.ncbi.nlm.nih.gov/protein/NRA20515.1?report=genbank&log$=prottop&blast_rank=41&RID=1HY1HUD3013) | 1.00E-49 |
| pentapeptide repeat-containing protein [Oceanospirillaceae bacterium] | 96% | 35.8 | [NQZ32382.1](https://www.ncbi.nlm.nih.gov/protein/NQZ32382.1?report=genbank&log$=prottop&blast_rank=42&RID=1HY1HUD3013) | 5.00E-47 |
| hypothetical protein A9R01_04950 ['Osedax' symbiont bacterium Rs2_46_30_T18] | 78% | 42.42 | [OUS37208.1](https://www.ncbi.nlm.nih.gov/protein/OUS37208.1?report=genbank&log$=prottop&blast_rank=43&RID=1HY1HUD3013) | 7.00E-47 |
| hypothetical protein OFPII_29990 [Osedax symbiont Rs1] | 98% | 36.29 | [EPJ45142.1](https://www.ncbi.nlm.nih.gov/protein/EPJ45142.1?report=genbank&log$=prottop&blast_rank=44&RID=1HY1HUD3013) | 1.00E-46 |
| hypothetical protein OFPI_08540 [Osedax symbiont Rs2] | 87% | 41.55 | [EPJ53986.1](https://www.ncbi.nlm.nih.gov/protein/EPJ53986.1?report=genbank&log$=prottop&blast_rank=45&RID=1HY1HUD3013) | 5.00E-46 |
| pentapeptide repeat-containing protein [Oceanospirillaceae bacterium] | 92% | 38.1 | [NRA15850.1](https://www.ncbi.nlm.nih.gov/protein/NRA15850.1?report=genbank&log$=prottop&blast_rank=46&RID=1HY1HUD3013) | 1.00E-45 |
| Qnr family pentapeptide repeat protein [Rahnella] | 85% | 34.11 | [WP_112288028.1](https://www.ncbi.nlm.nih.gov/protein/WP_112288028.1?report=genbank&log$=prottop&blast_rank=47&RID=1HY1HUD3013) | 1.00E-37 |
| Qnr family pentapeptide repeat protein [Rahnella aceris] | 85% | 34.11 | [WP_217217428.1](https://www.ncbi.nlm.nih.gov/protein/WP_217217428.1?report=genbank&log$=prottop&blast_rank=48&RID=1HY1HUD3013) | 2.00E-37 |
| Qnr family pentapeptide repeat protein [Rahnella aquatilis] | 85% | 33.18 | [WP_014333431.1](https://www.ncbi.nlm.nih.gov/protein/WP_014333431.1?report=genbank&log$=prottop&blast_rank=49&RID=1HY1HUD3013) | 4.00E-37 |
| Qnr family pentapeptide repeat protein [Rahnella sp. RFA10(1/100)] | 85% | 33.18 | [WP_129953898.1](https://www.ncbi.nlm.nih.gov/protein/WP_129953898.1?report=genbank&log$=prottop&blast_rank=50&RID=1HY1HUD3013) | 1.00E-36 |
| Qnr family pentapeptide repeat protein [Rahnella sp. CFA14(1/10)] | 85% | 33.18 | [WP_129991500.1](https://www.ncbi.nlm.nih.gov/protein/WP_129991500.1?report=genbank&log$=prottop&blast_rank=51&RID=1HY1HUD3013) | 2.00E-36 |
| Qnr family pentapeptide repeat protein [Rahnella bruchi] | 85% | 32.71 | [WP_120507780.1](https://www.ncbi.nlm.nih.gov/protein/WP_120507780.1?report=genbank&log$=prottop&blast_rank=52&RID=1HY1HUD3013) | 2.00E-36 |
| Qnr family pentapeptide repeat protein [Rahnella] | 85% | 31.78 | [WP_056777150.1](https://www.ncbi.nlm.nih.gov/protein/WP_056777150.1?report=genbank&log$=prottop&blast_rank=53&RID=1HY1HUD3013) | 3.00E-36 |
| Qnr family pentapeptide repeat protein [Rahnella bonaserana] | 85% | 33.18 | [WP_217171570.1](https://www.ncbi.nlm.nih.gov/protein/WP_217171570.1?report=genbank&log$=prottop&blast_rank=54&RID=1HY1HUD3013) | 3.00E-36 |
| Qnr family pentapeptide repeat protein [Rahnella sp. NRRL B-41462] | 85% | 33.18 | [WP_112197946.1](https://www.ncbi.nlm.nih.gov/protein/WP_112197946.1?report=genbank&log$=prottop&blast_rank=55&RID=1HY1HUD3013) | 9.00E-36 |
| Qnr family pentapeptide repeat protein [Rahnella victoriana] | 85% | 32.24 | [WP_095923911.1](https://www.ncbi.nlm.nih.gov/protein/WP_095923911.1?report=genbank&log$=prottop&blast_rank=56&RID=1HY1HUD3013) | 1.00E-35 |
| Qnr family pentapeptide repeat protein [Rahnella sp. BIGb0236] | 85% | 32.24 | [WP_133823073.1](https://www.ncbi.nlm.nih.gov/protein/WP_133823073.1?report=genbank&log$=prottop&blast_rank=57&RID=1HY1HUD3013) | 2.00E-35 |
| secreted effector protein PipB [Campylobacter jejuni] | 85% | 32.24 | [VTQ56603.1](https://www.ncbi.nlm.nih.gov/protein/VTQ56603.1?report=genbank&log$=prottop&blast_rank=58&RID=1HY1HUD3013) | 2.00E-35 |
| Qnr family pentapeptide repeat protein [Rahnella woolbedingensis] | 85% | 32.24 | [WP_120132835.1](https://www.ncbi.nlm.nih.gov/protein/WP_120132835.1?report=genbank&log$=prottop&blast_rank=59&RID=1HY1HUD3013) | 2.00E-35 |
| Qnr family pentapeptide repeat protein [Rahnella aquatilis] | 85% | 32.24 | [WP_047606448.1](https://www.ncbi.nlm.nih.gov/protein/WP_047606448.1?report=genbank&log$=prottop&blast_rank=60&RID=1HY1HUD3013) | 2.00E-35 |
| Qnr family pentapeptide repeat protein [Rahnella victoriana] | 85% | 32.24 | [WP_119825432.1](https://www.ncbi.nlm.nih.gov/protein/WP_119825432.1?report=genbank&log$=prottop&blast_rank=61&RID=1HY1HUD3013) | 2.00E-35 |
| Qnr family pentapeptide repeat protein [Rahnella ecdela] | 85% | 32.24 | [MBU9846306.1](https://www.ncbi.nlm.nih.gov/protein/MBU9846306.1?report=genbank&log$=prottop&blast_rank=62&RID=1HY1HUD3013) | 6.00E-35 |
| Qnr family pentapeptide repeat protein [Rahnella victoriana] | 85% | 31.78 | [WP_195817602.1](https://www.ncbi.nlm.nih.gov/protein/WP_195817602.1?report=genbank&log$=prottop&blast_rank=63&RID=1HY1HUD3013) | 1.00E-34 |
| hypothetical protein Q7S_00715 [Rahnella aquatilis HX2] | 85% | 31.78 | [AFE56410.1](https://www.ncbi.nlm.nih.gov/protein/AFE56410.1?report=genbank&log$=prottop&blast_rank=64&RID=1HY1HUD3013) | 3.00E-34 |
| Qnr family pentapeptide repeat protein [Rahnella] | 85% | 31.78 | [WP_013573489.1](https://www.ncbi.nlm.nih.gov/protein/WP_013573489.1?report=genbank&log$=prottop&blast_rank=65&RID=1HY1HUD3013) | 4.00E-34 |
| Qnr family pentapeptide repeat protein [Aeromonas veronii] | 83% | 33.01 | [WP_043821543.1](https://www.ncbi.nlm.nih.gov/protein/WP_043821543.1?report=genbank&log$=prottop&blast_rank=66&RID=1HY1HUD3013) | 5.00E-34 |
| Qnr family pentapeptide repeat protein [Aeromonas sp. FDAARGOS 1411] | 83% | 33.01 | [WP_216960339.1](https://www.ncbi.nlm.nih.gov/protein/WP_216960339.1?report=genbank&log$=prottop&blast_rank=67&RID=1HY1HUD3013) | 6.00E-34 |
| Qnr family pentapeptide repeat protein [Enterobacteriaceae bacterium Kacie_13] | 85% | 31.31 | [QLK63365.1](https://www.ncbi.nlm.nih.gov/protein/QLK63365.1?report=genbank&log$=prottop&blast_rank=68&RID=1HY1HUD3013) | 6.00E-34 |
| Qnr family pentapeptide repeat protein [Rahnella inusitata] | 85% | 31.31 | [WP_112167471.1](https://www.ncbi.nlm.nih.gov/protein/WP_112167471.1?report=genbank&log$=prottop&blast_rank=69&RID=1HY1HUD3013) | 7.00E-34 |
| Qnr family pentapeptide repeat protein [Aeromonas fluvialis] | 83% | 33.01 | [WP_042010234.1](https://www.ncbi.nlm.nih.gov/protein/WP_042010234.1?report=genbank&log$=prottop&blast_rank=70&RID=1HY1HUD3013) | 7.00E-34 |
| Qnr protein [Vibrio maritimus] | 88% | 32.58 | [GAL35640.1](https://www.ncbi.nlm.nih.gov/protein/GAL35640.1?report=genbank&log$=prottop&blast_rank=71&RID=1HY1HUD3013) | 8.00E-34 |
| Qnr family pentapeptide repeat protein [Salmonella enterica] | 82% | 34.3 | [EAM2074469.1](https://www.ncbi.nlm.nih.gov/protein/EAM2074469.1?report=genbank&log$=prottop&blast_rank=72&RID=1HY1HUD3013) | 1.00E-33 |
| TPA: hypothetical protein [Gammaproteobacteria bacterium] | 91% | 34.78 | [HAK52355.1](https://www.ncbi.nlm.nih.gov/protein/HAK52355.1?report=genbank&log$=prottop&blast_rank=73&RID=1HY1HUD3013) | 2.00E-33 |
| pentapeptide repeat-containing protein [Photorhabdus temperata subsp. temperata M1021] | 84% | 33.33 | [EQB99381.1](https://www.ncbi.nlm.nih.gov/protein/EQB99381.1?report=genbank&log$=prottop&blast_rank=74&RID=1HY1HUD3013) | 2.00E-33 |
| Qnr family pentapeptide repeat protein [Salmonella enterica] | 82% | 33.82 | [EAV2671145.1](https://www.ncbi.nlm.nih.gov/protein/EAV2671145.1?report=genbank&log$=prottop&blast_rank=75&RID=1HY1HUD3013) | 2.00E-33 |
| Qnr family pentapeptide repeat protein [Photorhabdus temperata] | 84% | 33.33 | [WP_023045781.1](https://www.ncbi.nlm.nih.gov/protein/WP_023045781.1?report=genbank&log$=prottop&blast_rank=76&RID=1HY1HUD3013) | 3.00E-33 |
| Qnr family pentapeptide repeat protein [Rahnella aquatilis] | 85% | 31.31 | [WP_126125286.1](https://www.ncbi.nlm.nih.gov/protein/WP_126125286.1?report=genbank&log$=prottop&blast_rank=77&RID=1HY1HUD3013) | 3.00E-33 |
| hypothetical protein [Gammaproteobacteria bacterium] | 91% | 34.78 | [MAV26548.1](https://www.ncbi.nlm.nih.gov/protein/MAV26548.1?report=genbank&log$=prottop&blast_rank=78&RID=1HY1HUD3013) | 3.00E-33 |
| Qnr family pentapeptide repeat protein [Rouxiella chamberiensis] | 85% | 31.31 | [KAB8306065.1](https://www.ncbi.nlm.nih.gov/protein/KAB8306065.1?report=genbank&log$=prottop&blast_rank=79&RID=1HY1HUD3013) | 3.00E-33 |
| Qnr family pentapeptide repeat protein [Aeromonas veronii] | 83% | 32.54 | [WP_064339168.1](https://www.ncbi.nlm.nih.gov/protein/WP_064339168.1?report=genbank&log$=prottop&blast_rank=80&RID=1HY1HUD3013) | 3.00E-33 |
| Qnr family pentapeptide repeat protein [Salmonella enterica subsp. arizonae] | 82% | 33.82 | [EDZ3529595.1](https://www.ncbi.nlm.nih.gov/protein/EDZ3529595.1?report=genbank&log$=prottop&blast_rank=81&RID=1HY1HUD3013) | 4.00E-33 |
| Qnr family pentapeptide repeat protein [Aeromonas veronii] | 83% | 32.06 | [WP_164535829.1](https://www.ncbi.nlm.nih.gov/protein/WP_164535829.1?report=genbank&log$=prottop&blast_rank=82&RID=1HY1HUD3013) | 4.00E-33 |
| Qnr family pentapeptide repeat protein [Aeromonas veronii] | 83% | 32.54 | [WP_236269117.1](https://www.ncbi.nlm.nih.gov/protein/WP_236269117.1?report=genbank&log$=prottop&blast_rank=83&RID=1HY1HUD3013) | 4.00E-33 |
| Qnr family pentapeptide repeat protein [Ewingella americana] | 85% | 31.63 | [WP_140474869.1](https://www.ncbi.nlm.nih.gov/protein/WP_140474869.1?report=genbank&log$=prottop&blast_rank=84&RID=1HY1HUD3013) | 4.00E-33 |
| fluoroquinolone resistance protein [Ewingella americana] | 85% | 31.31 | [PKB89033.1](https://www.ncbi.nlm.nih.gov/protein/PKB89033.1?report=genbank&log$=prottop&blast_rank=85&RID=1HY1HUD3013) | 5.00E-33 |
| Qnr family pentapeptide repeat protein [Aeromonas veronii] | 83% | 33.01 | [WP_181269743.1](https://www.ncbi.nlm.nih.gov/protein/WP_181269743.1?report=genbank&log$=prottop&blast_rank=86&RID=1HY1HUD3013) | 5.00E-33 |
| Qnr family pentapeptide repeat protein [Aeromonas allosaccharophila] | 83% | 32.06 | [WP_163128616.1](https://www.ncbi.nlm.nih.gov/protein/WP_163128616.1?report=genbank&log$=prottop&blast_rank=87&RID=1HY1HUD3013) | 6.00E-33 |
| Qnr family pentapeptide repeat protein [Rahnella sp. ERMR1:05] | 85% | 30.84 | [WP_104923753.1](https://www.ncbi.nlm.nih.gov/protein/WP_104923753.1?report=genbank&log$=prottop&blast_rank=88&RID=1HY1HUD3013) | 6.00E-33 |
| Qnr family pentapeptide repeat protein [Ewingella americana] | 85% | 31.31 | [WP_154145731.1](https://www.ncbi.nlm.nih.gov/protein/WP_154145731.1?report=genbank&log$=prottop&blast_rank=89&RID=1HY1HUD3013) | 6.00E-33 |
| Qnr family pentapeptide repeat protein [Ewingella americana] | 85% | 31.31 | [WP_034794010.1](https://www.ncbi.nlm.nih.gov/protein/WP_034794010.1?report=genbank&log$=prottop&blast_rank=90&RID=1HY1HUD3013) | 6.00E-33 |
| Qnr family pentapeptide repeat protein [Aeromonas veronii] | 83% | 33.01 | [WP_139442306.1](https://www.ncbi.nlm.nih.gov/protein/WP_139442306.1?report=genbank&log$=prottop&blast_rank=91&RID=1HY1HUD3013) | 7.00E-33 |
| Qnr family pentapeptide repeat protein [Salmonella enterica] | 82% | 33.82 | [EHJ4202533.1](https://www.ncbi.nlm.nih.gov/protein/EHJ4202533.1?report=genbank&log$=prottop&blast_rank=92&RID=1HY1HUD3013) | 7.00E-33 |
| quinolone resistance pentapeptide repeat protein QnrB80 [Citrobacter freundii] | 78% | 35.2 | [WP_065825703.1](https://www.ncbi.nlm.nih.gov/protein/WP_065825703.1?report=genbank&log$=prottop&blast_rank=93&RID=1HY1HUD3013) | 8.00E-33 |
| Qnr family pentapeptide repeat protein [Aeromonas veronii] | 83% | 33.01 | [WP_139477759.1](https://www.ncbi.nlm.nih.gov/protein/WP_139477759.1?report=genbank&log$=prottop&blast_rank=94&RID=1HY1HUD3013) | 8.00E-33 |
| Qnr family pentapeptide repeat protein [Aeromonas veronii] | 83% | 33.01 | [WP_213387905.1](https://www.ncbi.nlm.nih.gov/protein/WP_213387905.1?report=genbank&log$=prottop&blast_rank=95&RID=1HY1HUD3013) | 9.00E-33 |
| Qnr family pentapeptide repeat protein [Aeromonas veronii] | 83% | 33.01 | [WP_201958967.1](https://www.ncbi.nlm.nih.gov/protein/WP_201958967.1?report=genbank&log$=prottop&blast_rank=96&RID=1HY1HUD3013) | 9.00E-33 |
| Qnr family pentapeptide repeat protein [Rahnella] | 78% | 33.16 | [WP_235174222.1](https://www.ncbi.nlm.nih.gov/protein/WP_235174222.1?report=genbank&log$=prottop&blast_rank=97&RID=1HY1HUD3013) | 1.00E-32 |
| Qnr family pentapeptide repeat protein [Aeromonas veronii] | 86% | 31.94 | [WP_219265134.1](https://www.ncbi.nlm.nih.gov/protein/WP_219265134.1?report=genbank&log$=prottop&blast_rank=98&RID=1HY1HUD3013) | 1.00E-32 |
| Qnr family pentapeptide repeat protein [Rahnella ecdela] | 78% | 33.16 | [WP_238485545.1](https://www.ncbi.nlm.nih.gov/protein/WP_238485545.1?report=genbank&log$=prottop&blast_rank=99&RID=1HY1HUD3013) | 1.00E-32 |
| Qnr family pentapeptide repeat protein [Aeromonas veronii] | 83% | 33.01 | [WP_139747010.1](https://www.ncbi.nlm.nih.gov/protein/WP_139747010.1?report=genbank&log$=prottop&blast_rank=100&RID=1HY1HUD3013) | 1.00E-32 |

Table S6. Mutations from speirobactin-resistant *M. tuberculosis* mutants. RA: read alignment. JC: new junction.

| **Mutant No.1** |  |  |  |  |  |  |
| --- | --- | --- | --- | --- | --- | --- |
| **evidence** | **position** | **mutation** | **annotation** | **gene** | **description** | **Essential** |
| RA | 7567 | A→G | D89G (GAC→GGC) | gyrA → | DNA gyrase (subunit A) GyrA (DNA topoisomerase (ATP‑hydrolysing)) (DNA topoisomerase II) (type II DNA topoisomerase) | Y |
| RA | 2596695 | T→G | L121R (CTC→CGC) | Rv2324 → | Probable transcriptional regulatory protein (probably AsnC‑family) | N |
| RA | 3250276 | Δ1 bp | coding (4832/5631 nt) | ppsA → | Phenolpthiocerol synthesis type‑I polyketide synthase PpsA | N |
| RA | 3689763 | G→A | A392A (GCC→GCT) | lpdA ← | NAD(P)H quinone reductase LpdA | Conditional |
| JC | 3691003 | 58 bp x 2 | duplication | lpdA ← / → Rv3304 | NAD(P)H quinone reductase LpdA/Conserved protein | Conditional |
| JC | 3779699 | (GCAACGGTG)3→4 | coding (1132/1767 nt) | PE_PGRS51 → | PE‑PGRS family protein PE_PGRS51 | N |
|  |  |  |  |  |  |  |
| **Mutant No. 2** |  |  |  |  |  |  |
| **evidence** | **position** | **mutation** | **annotation** | **gene** | **description** | **Essential** |
| RA | 7604 | G→C | Q101H (CAG→CAC) | gyrA → | DNA gyrase (subunit A) GyrA (DNA topoisomerase (ATP‑hydrolysing)) (DNA topoisomerase II) (type II DNA topoisomerase) | Y |
| RA | 839123 | A→G | R225G (AGG→GGG) | PE_PGRS10 → | PE‑PGRS family protein PE_PGRS10 | N |
| RA | 839129 | C→G | R227G (CGC→GGC) | PE_PGRS10 → | PE‑PGRS family protein PE_PGRS10 | N |
| RA | 839348 | A→G | S300G (AGC→GGC) | PE_PGRS10 → | PE‑PGRS family protein PE_PGRS10 | N |
| RA | 840496 | C→G | G682G (GGC→GGG) | PE_PGRS10 → | PE‑PGRS family protein PE_PGRS10 | N |
| RA | 2596749 | T→C | L139P (CTG→CCG) | Rv2324 → | Probable transcriptional regulatory protein (probably AsnC‑family) | N |
| RA | 3251766 | #CALC! | coding (695/4617 nt) | ppsB → | Phenolpthiocerol synthesis type‑I polyketide synthase PpsB | N |
| JC | 3690953 | +AGC | intergenic (‑15/‑188) | lpdA ← / → Rv3304 | NAD(P)H quinone reductase LpdA/Conserved protein | Conditional |
| JC | 3691003 | 58 bp x 2 | duplication | lpdA ← / → Rv3304 | NAD(P)H quinone reductase LpdA/Conserved protein | Conditional |
| JC | 3779699 | (GCAACGGTG)3→4 | coding (1132/1767 nt) | PE_PGRS51 → | PE‑PGRS family protein PE_PGRS51 | N |
|  |  |  |  |  |  |  |
| **Mutant No. 3** |  |  |  |  |  |  |
| **evidence** | **position** | **mutation** | **annotation** | **gene** | **description** | **Essential** |
| RA | 7567 | A→G | D89G (GAC→GGC) | gyrA → | DNA gyrase (subunit A) GyrA (DNA topoisomerase (ATP‑hydrolysing)) (DNA topoisomerase II) (type II DNA topoisomerase) | Y |
| RA | 838990 | C→G | A180A (GCC→GCG) | PE_PGRS10 → | PE‑PGRS family protein PE_PGRS10 | N |
| RA | 839123 | A→G | R225G (AGG→GGG) | PE_PGRS10 → | PE‑PGRS family protein PE_PGRS10 | N |
| RA | 839129 | C→G | R227G (CGC→GGC) | PE_PGRS10 → | PE‑PGRS family protein PE_PGRS10 | N |
| RA | 839348 | A→G | S300G (AGC→GGC) | PE_PGRS10 → | PE‑PGRS family protein PE_PGRS10 | N |
| RA | 2596695 | T→G | L121R (CTC→CGC) | Rv2324 → | Probable transcriptional regulatory protein (probably AsnC‑family) | N |
| RA | 3250276 | Δ1 bp | coding (4832/5631 nt) | ppsA → | Phenolpthiocerol synthesis type‑I polyketide synthase PpsA | N |
| RA | 3689763 | G→A | A392A (GCC→GCT) | lpdA ← | NAD(P)H quinone reductase LpdA | Conditional |
| JC | 3691003 | 58 bp x 2 | duplication | lpdA ← / → Rv3304 | NAD(P)H quinone reductase LpdA/Conserved protein | Conditional |
| JC | 3779699 | (GCAACGGTG)3→4 | coding (1132/1767 nt) | PE_PGRS51 → | PE‑PGRS family protein PE_PGRS51 | N |
| RA | 3940802 | A→G | N396D (AAC→GAC) | PE_PGRS55 → | PE‑PGRS family protein PE_PGRS55 | N |
|  |  |  |  |  |  |  |
| **Mutant No. 4** |  |  |  |  |  |  |
| **evidence** | **position** | **mutation** | **annotation** | **gene** | **description** | **Essential** |
| RA | 7684 | G→A | R128K (AGG→AAG) | gyrA → | DNA gyrase (subunit A) GyrA (DNA topoisomerase (ATP‑hydrolysing)) (DNA topoisomerase II) (type II DNA topoisomerase) | Y |
| RA | 1982322 | Δ1 bp | coding (2454/3162 nt) | PPE24 ← | PPE family protein PPE24 | N |
| RA | 2596695 | T→G | L121R (CTC→CGC) | Rv2324 → | Probable transcriptional regulatory protein (probably AsnC‑family) | N |
| RA | 3250276 | Δ1 bp | coding (4832/5631 nt) | ppsA → | Phenolpthiocerol synthesis type‑I polyketide synthase PpsA | N |
| RA | 3689763 | G→A | A392A (GCC→GCT) | lpdA ← | NAD(P)H quinone reductase LpdA | Conditional |
| RA | 3779699 | (GCAACGGTG)3→4 | coding (1132/1767 nt) | PE_PGRS51 → | PE‑PGRS family protein PE_PGRS51 | N |

Table S7. Upregulated genes shared by speirobactin, ciprofloxacin and moxifloxacin from RNA-seq studies (log2fc >1, p-value ≤0.05).

| **Locus tag** | **Gene** | **Description** |
| --- | --- | --- |
| Rv2885c | Rv2885c | Transposase |
| Rv3190A | Rv3190A | Unknown |
| Rv2737c | RecA | Homologous recombination |
| Rv1588c | Rv1588c | Unknown |
| Rv3517 | Rv3517 | Unknown |
| Rv2979c | Rv2979c | Resolvase |
| Rv3202c | Rv3202c | ATP-dependent helicase; induced by oxidative stress |
| Rv1286 | CysN/CysC | Probable bifunctional enzyme CysN/CysC: sulfate adenyltransferase (subunit 1) + adenylylsulfate kinase |
| Rv2734 | Rv2734 | Unknown |
| Rv1316c | Ogt | Methylated-DNA--protein-cysteine methyltransferase; Repair of alkylated guanine in DNA |
| Rv2886c | Rv2886c | Probable resolvase |
| Rv1148c | Rv1148c | Induced by DNA damage |
| Rv2191 | Rv2191 | DNA replication, Mismatch repair, Homologous recombination; RecA indep induction in response to MitoC treatment; expressed during chronic infection in rabbits |
| Rv2100 | Rv2100 | Unknown |
| Rv2719c | Rv2719c | Possible conserved membrane protein |
| Rv1317c | AlkA | Base excision repair |
| Rv0095c | Rv0095c | Uncharacterized protein |
| Rv1945 | Rv1945 | Unknown |
| Rv3201c | PcrA | Nucleotide excision repair, Mismatch repair; regulates recombination displacing recA |

Table S8. Downregulated genes shared by speirobactin, ciprofloxacin and moxifloxacin from RNA-seq studies (log2fc >1, p-value ≤0.05).

| **Locus tag** | **Gene** | **Description** |
| --- | --- | --- |
| Rv0970 | Rv0970 | Probable conserved integral membrane protein |
| Rv1709 | ScpA | Possible segregation and condensation protein ScpA |
| Rv1451 | CtaB | Oxidative phosphorylation; Porphyrin and chlorophyll metabolism |
| Rv1714 | Rv1714 | Probable oxidoreductase |
| Rv1642 | RpmI | Ribosome |
| Rv1415 | RibA2 | Riboflavin metabolism; Folate biosynthesis |
| Rv1087 | PE_PGRS21 | Unknown |
| Rv0956 | PurN | Purine metabolism; One carbon pool by folate |
| Rv0899 | OmpA | Probable oxidoreductase |
| Rv1143 | Mcr | Probable alpha-methylacyl-CoA racemase Mcr (2-methylacyl-CoA racemase) (2-arylpropionyl-CoA epimerase ) |
| Rv1395 | Rv1395 | Transcriptional regulatory protein |
| Rv1375 | Rv1375 | Unknown |
| Rv1188 | Rv1188 | Arginine and proline metabolism |
| Rv0988 | Rv0988 | Possible conserved exported protein |
| Rv1631 | CoaE | Pantothenate and CoA biosynthesis |
| Rv1612 | TrpB | Glycine, serine and threonine metabolism; Phenylalanine, tyrosine and tryptophan biosynthesis |
| Rv0977 | PE_PGRS16 | unknown |
| Rv0967 | CsoR | Copper-sensitive operon repressor CsoR |
| Rv1408 | Rpe | Pentose phosphate pathway; Pentose and glucuronate interconversions |
| Rv1659 | ArgH | Alanine, aspartate and glutamate metabolism; Arginine biosynthesis |
| Rv1073 | Rv1073 | Unknown |
